# Ferric coagulants produced from basic oxygen furnace sludge—Part II: optimization of ferrous sulfate production and application of ferric coagulants in water treatment

**DOI:** 10.1007/s11356-026-37973-9

**Published:** 2026-07-03

**Authors:** Luisa Cardoso Maia, Grazielle Rocha dos Santos, Andressa Rezende Pereira, Yasmim Arantes da Fonseca, Leandro Vinícius Alves Gurgel, Cornélio de Freitas Carvalho

**Affiliations:** 1 Environmental Engineering Graduate Program, School of Mines, Federal University of Ouro Preto, Universitário Morro Do Cruzeiro, Campus Morro do Cruzeiro, Rua Nove, s/n, Bauxita, 35402-163, Ouro Preto, Minas Gerais, Brazil; 2 Department of Chemistry, Institute of Exact and Biological Sciences, Federal University of Ouro Preto, Universitário Morro Do Cruzeiro, Campus Morro do Cruzeiro, Rua Quatro, 786, Bauxita, 35402-136, Ouro Preto, Minas Gerais, Brazil

**Keywords:** BOF sludge, Iron recovery, Recycling, Water treatment, Economic analysis

## Abstract

A novel process was employed to produce ferric coagulants using iron recovered from the coarse and fine fractions of basic oxygen furnace (BOF) sludge. The FeSO_4_ obtained as an intermediate product was converted to Fe_2_(SO_4_)_3_ (final product), which was used as a coagulant in water treatment. The iron contents of the coarse and fine BOF sludge fractions were 82.51 ± 0.02% and 45.6 ± 0.5% (dry basis), respectively. Iron was recovered from the sludge fractions by acid leaching with H_2_SO_4_ solution, followed by the addition of ethanol to the leachate to crystallize FeSO_4_, from which ferric coagulants were obtained by oxidation reaction with H_2_O_2_. The performance of the coagulants was evaluated using the jar test method. The optimum condition for the production of FeSO_4_ from the fine fraction of the BOF sludge was established using a desirability function. This resulted in the formation of 13.1 ± 0.2 g of FeSO_4_‧7H_2_O from 7.00 g of waste, with a high yield of 91 ± 1%, using 13% (v/v) H_2_SO_4_ solution, a leaching time of 140 min, and 120 mL of ethanol. The phase composition of the FeSO_4_ was investigated by X-ray diffraction (XRD), thermogravimetric analysis (TGA), scanning electron microscopy (SEM), and energy dispersive X-ray spectroscopy (EDX). The Fe_2_(SO_4_)_3_ produced from the coarse and fine fractions of BOF sludge achieved high turbidity removals (95 ± 1% and 94.1 ± 0.9%, respectively), demonstrating its potential as a coagulant. Performance was compared with a commercial coagulant (polyaluminum chloride), confirming competitive efficiency (97 ± 1%). In addition, bench-scale production costs of ferric coagulants were estimated, and a sensitivity analysis was conducted to assess economic feasibility.

**Supplementary Information:**

The online version contains supplementary material available at 10.1007/s11356-026-37973-9.

## Introduction

Various byproducts are generated in the steel industry, including slag, sludge, scale, and dust (Matino et al. 2017). One of these is basic oxygen furnace (BOF) sludge, which is separated according to particle size into coarse and fine fractions that both contain iron as the predominant element. The fine fraction is composed of particles removed by the gas cleaning system (Cantarino et al. 2012). This waste represents the largest fraction of BOF sludge, where 70–80% consists of fine particles smaller than 38 µm (dos Santos et al. 2015; Földi et al. 2014). As reported by Cantarino et al. (2012), approximately 20 kg of the fine fraction of BOF sludge is produced per ton of crude steel.

There is no direct reuse for the fine fraction of BOF sludge, due to the presence of contaminants such as zinc and alkali, so it is considered a waste that is problematic to manage (de Lima et al. 2013; dos Santos et al. 2015; Xia et al. 2015). These contaminants, especially zinc, contribute to the formation of a crust on the refractory wall of the blast furnace (Das et al. 2007; Ma 2016; Xie et al. 2017). The fine fraction of BOF sludge has commonly been buried in landfills for nonhazardous waste (de Lima et al. 2013). However, this waste contains metals of economic interest, including a high content of iron, mainly in the form of iron oxides (Xia et al. 2015).

An attractive option for the reuse of BOF sludge is the recovery of valuable metals for obtaining value-added products (Purohit and Satapathy 2016). Studies have proposed new routes for recycling the fine fraction of BOF sludge, but all require the prior removal of zinc (Cantarino et al. 2012; Kelebek et al. 2004; Kukurugya et al. 2015; Lobato et al. 2015; Trung et al. 2011). Other possible applications include use in asphalt mixtures (Xie et al. 2017), production of briquettes (de Oliveira and Bagatini 2019; Xia et al. 2015), and adsorption of contaminants (dos Santos et al. 2015). There are techniques for processing steel waste that allow the recovery of metals, such as acid leaching (Das et al. 2007; Siedlecka 2020; Xie et al. 2017), which can enable the recovery of useful elements, reducing costs and the environmental impact of steelmaking operations (Siedlecka 2020).

Recycling of the coarse and fine fractions of BOF sludge generates ferrous sulfate as a byproduct, which can be used as an intermediate in the production of ferric coagulant employed in water treatment plants (WTPs). In this way, the recycling of BOF sludge contributes to reducing the environmental impact of the steel industry by producing an efficient coagulant. Beyond this application, ferrous sulfate produced from BOF sludge could also serve as a precursor for FePO_4_ synthesis, which is a potential material for LiFePO_4_ cathodes in lithium-ion batteries, widely used in electric vehicles (Qiu et al. 2025).

The proposed process further presents economic advantages compared to other methods suggested for recycling steelmaking waste (de Buzin et al. 2014; Trung et al. 2011), while introducing new applications for the byproducts generated from both BOF sludge fractions. Acid leaching has the additional advantage of not requiring pre-drying of BOF sludge, unlike other approaches. Moreover, ferrous sulfate crystallization can be performed using ethanol (EtOH), which is available from renewable sources (Abu Tayeh et al. 2014; Geddes et al. 2011; Zhuang et al. 2016), reinforcing the sustainability of the process.

Despite these advantages, some environmental aspects should be noted. The use of ferric coagulant in water treatment inevitably generates sludge; however, ferric sludge from conventional treatment has been shown to be manageable within integrated wastewater and sludge treatment systems and can also serve as a source of iron recovery (Nayeri and Mousavi 2022; Wu et al. 2023). In addition, the use of sulfuric acid, ethanol, and hydrogen peroxide introduces upstream environmental burdens, meaning that the net benefit of waste valorization depends on reagent recovery, process optimization, and comprehensive life-cycle assessment prior to scale-up (Klimtová et al. 2025; Sefiddashti et al. 2026).

An alternative environmental interest is the use of titanium white waste acid (TWWA), a byproduct from the sulfuric acid method of TiO_2_ production, as a leaching agent for metal recovery from industrial wastes (Qiu et al. 2025). This approach, often described as “treating waste with waste,” can potentially reduce production costs by replacing commercial acids, although the impurities in TWWA pose challenges for purification steps, as highlighted in recent studies on pyrite residue (Tian 2024).

In the first part of this study (Maia et al. 2020), a new recycling process was proposed for the coarse fraction of BOF sludge, focusing on iron recovery through acid leaching and ferrous sulfate crystallization within the same solution. However, the fine fraction of BOF sludge, which has distinct physicochemical characteristics and presents management challenges (Binnemans et al. 2020), was not addressed. The study extends the recycling approach to the fine fraction by employing multivariate optimization to determine the optimal conditions for ferrous sulfate production. This study introduces a significant technological advancement: the conversion of recovered iron into ferric coagulants, demonstrating its applicability in water treatment. Additionally, a preliminary cost analysis provided initial insights into the economic feasibility of the process. These advancements—addressing the fine fraction, validating environmental applications, and assessing preliminary economic viability—represent substantial progress beyond that of the first part of this study.

## Material and methods

### Material

The coarse and fine BOF sludge fractions were provided by a steel company in Minas Gerais state, Brazil, and were dried before use. Ethanol (99 wt.%), sulfuric acid (97%), and hydrogen peroxide (35 wt.%) were purchased from Neon (Brazil). Polyaluminum chloride (PAC) was purchased from QuimisulSC, Santa Catarina state, Brazil (18 wt.%). Calcium hydroxide was purchased from Miika (Brazil). The surface water used in the jar tests was collected in Ouro Preto (Minas Gerais state, Brazil).

### Characterization of the BOF sludge fine fraction

Elemental analysis of the BOF sludge fine fraction was performed by X-ray fluorescence (XRF) spectroscopy (model Zetium, Malvern Panalytical), using a pressed sample. The loss on ignition (LOI) was measured at 1020 °C. The chemical composition of the fine sludge fraction was determined by analysis of a digested sample using inductively coupled plasma optical emission spectroscopy (ICP-OES) (model Optima 8300, PerkinElmer). The total iron and Fe(II) contents were determined according to the methods described by the Brazilian Association of Technical Standards (ABNT 2003) and Mendham et al. (2000), respectively.

### Production of ferrous sulfate from the fine fraction of BOF sludge

The procedure to obtain ferrous sulfate heptahydrate, involving acid leaching of BOF sludge followed by crystallization of ferrous sulfate, was adapted from Maia et al. (2020). Leaching of the fine fraction was carried out in Erlenmeyer flasks at 25.0 ± 0.1 °C using an orbital shaker-incubator (model TE-424, Tecnal) set to 200 rpm, with 50.0 ± 0.1 mL of sulfuric acid solution. After leaching, the pulp was filtered through a glass fiber filter in a Büchner funnel, separating the iron-rich leachate from the residual sludge. Crystallization of ferrous sulfate was induced by adding EtOH to the leachate, and the resulting crystals were recovered by vacuum filtration and dried at 30.0 ± 0.1 °C in a vacuum oven.

Multivariate optimization of ferrous sulfate production employed a 2^4^ experimental design, for initial screening, followed by a Doehlert experimental design (DED), for optimization of the conditions. The factors investigated in the optimization experiments were the H_2_SO_4_ solution concentration (*c*, % v/v), leaching time (*t*, min), waste amount (*w*, g), and EtOH volume (*e*, mL). The response variables were the mass of ferrous sulfate heptahydrate (FeSO_4_·7H_2_O, g) and the process yield (yield, %), which was calculated considering the theoretical mass of ferrous sulfate heptahydrate, based on the Fe(II) content of the BOF sludge fine fraction. The Student’s *t* distribution and replicates of the center point were used to determine the significant factors (*p* < 0.05) and to estimate the error, respectively. All experimental runs were randomized to minimize bias. Each condition was performed in duplicate, meaning independent experiments on different days with freshly prepared solutions, ensuring reproducibility and reliability of the results. The experimental data were analyzed using Statistica software (version 10.0, StatSoft, Inc.).

To evaluate impurities in the ferrous sulfate, the leachate obtained under the optimal condition for ferrous sulfate production was characterized by ICP-OES (model Optima 8300, PerkinElmer).

### Characterization of the ferrous sulfate crystals

X-ray diffraction analysis (XRD) of the ferrous sulfate produced from the BOF sludge fine fraction employed a diffractometer (model XRD-7000, Shimadzu) with a graphite monochromator and a nickel filter. The instrument was operated with Cu Kα radiation (*λ* = 1.5406 Å), at 30 mA and 40 kV. The surface of the ferrous sulfate was investigated using a scanning electron microscope (model FEI Quanta 650 FEG, Thermo Fisher) coupled to an energy dispersive X-ray spectroscopy analyzer (model Quantax, Bruker) equipped with an XFlash 4030 silicon drift detector (SDD). Thermal analysis of the ferrous sulfate employed a thermogravimetric analyzer (model DTG-60, Shimadzu), with heating at a rate of 10 °C min^−1^, under an atmosphere of N_2_ at a flow rate of 20 mL min^−1^.

### Production of ferric coagulants from the coarse and fine BOF sludge fractions

Ferric coagulants were produced from ferrous sulfate obtained from the coarse (Maia et al. 2020) and fine fractions of the BOF sludge (denoted FS-I and FS-II) by oxidation with hydrogen peroxide. Prior to oxidation, the ferrous sulfate crystals were solubilized in an acid solution containing 7.5 mL of distilled water and 0.25 mL of sulfuric acid (97 wt.%). In the oxidation step, 5.00 g of ferrous sulfate heptahydrate was reacted with hydrogen peroxide volumes of 0.20, 1.0, and 2.0 mL, corresponding to FeSO_4_·7H_2_O:H_2_O_2_ ratios of 1:0.04, 1:0.2, and 1:0.4 (w/v). The resulting ferric coagulants, FC-I and FC-II, were characterized by ICP-OES after solubilization in distilled water. Total iron content was determined according to ABNT (2003), while Fe(II) and Fe(III) contents were measured following the procedure described by Mendham et al. (2000).

### Evaluation of the ferric coagulants in water clarification

The FC-I and FC-II coagulants were evaluated using a jar test system (Nova Ética, Brazil) to simulate the clarification process employed in most WTPs. The performance of these ferric coagulants was evaluated and compared with a commercial coagulant, PAC, under the same experimental conditions. The characteristics of the surface water used in the experiments are shown in Table 1. Turbidity and pH were measured using a portable turbidimeter (model 2100Q, Hach) and pH probe (model HQ40D, Hach), respectively. The hydraulic conditions in the tests were set according to the Brazilian Association of Technical Standards (ABNT 1992), as follows: rapid mixing time (*T*_rm_) of 10 s and velocity gradient (*G*_rm_) of 800 s^−1^; flocculation time (*T*_f_) of 20 min and velocity gradient (*G*_f_) of 35 s^−1^; and settling velocity of 1.74 cm min^−1^. Jar test experiments were conducted using coagulant doses from 5 to 30 mg L^−1^. Each condition was performed in duplicate, and the results are presented as mean values with standard deviation. The tests were performed at pH values between 6.2 and 6.9 for ferric-based coagulants, and between 7.5 and 7.9 for PAC, with sweep coagulation identified as the predominant removal mechanism. The coagulation pH was adjusted by adding calcium hydroxide. The evaluation of coagulant performance was based on turbidity removal, which is the primary parameter used in conventional Brazilian water treatment plants to monitor and control coagulation efficiency, reflecting the operational practices in treatment. The amount of coagulant that resulted in the highest removal of turbidity was considered the optimal dose. Additionally, the safety of coagulants in water treatment was evaluated through a compliance analysis with EN standards concerning impurities in coagulants.

**Table 1:** Characteristics of the high turbidity surface water

Parameter	Mean value
Turbidity (NTU)	214 ± 2
Apparent color (uH)	373 ± 50
True color (uH)	14 ± 2
Temperature (°C)	18
pH	7.5 ± 0.1
Total alkalinity (mg CaCO_3_ L^−1^)	13 ± 1

### Cost estimation for the production of ferrous sulfate and ferric coagulant from the fine and coarse fractions of BOF sludge

Estimates were made of the costs of bench-scale production of ferrous sulfate (FS-I and FS-II) and ferric coagulants (FC-I and FC-II) from the coarse and fine fractions of the BOF sludge. The estimates were based on the optimized conditions for the production of FS-I, determined in the first part of this study (Maia et al. 2020), and FS-II, determined in the present study. For production of the ferric coagulants, the FeSO_4_·7H_2_O:H_2_O_2_ ratio of 1:0.4 (w/v) was adopted, since this condition provided the highest oxidation efficiency in the present study.

The aim of this analysis was to obtain an economic parameter for comparing the products derived from the coarse fraction (FS-I and FC-I) and the fine fraction (FS-II and FC-II) of the BOF sludge. The cost estimation considered the prices of reagents, water, and electricity applicable to each stage of the process. The reagent prices were obtained from local suppliers (Neon Comercial 2021). The water price used in the calculations was US$ 0.002 L^−1^, as charged by the water supply company in Minas Gerais state (Copasa 2020). Electricity costs were based on the average energy tariff (US$ 0.11 kWh^−1^) charged by the energy supply company in Minas Gerais state (Cemig 2021). In addition, it was considered that 80% of the EtOH (Vigânico 2014) and sulfuric acid (Amaral et al. 2018) used in the production of ferrous sulfate could be recovered and reused in subsequent cycles. A sensitivity analysis was also performed by varying the recovery percentages of EtOH and sulfuric acid between 0 and 95% to evaluate the impact of reagent reuse on the economic feasibility of the process.

## Results and discussion

### Characterization of the BOF sludge fine fraction

The XRF analysis of the BOF sludge fine fraction showed that the elemental composition was dominated by iron and calcium (Table 2). Other elements present, related to the steel production process, included magnesium, zinc, manganese, and aluminum. Unlike the coarse fraction of the BOF sludge, reported in the first part of this study (Maia et al. 2020), the LOI analysis suggested that part of the material was in the oxidized form, as also reported by Cantarino et al. (2012). The chemical composition of the BOF sludge fine fraction is shown in Table 3. As observed in the XRF analysis, the major elements present were iron (47.8 ± 0.0%) and calcium (9.3 ± 0.0%).

**Table 2:** Elemental analysis of the BOF sludge fine fraction by X-ray fluorescence (XRF)

Composition	Elements
High quantities	Fe, Ca
Low quantities	Si, Mg, Zn, Mn, Al, Na, S, K, P, Cl
Trace quantities	Ti, V, Cr, Sr, Pb, Cu
Loss on ignition (LOI)	6.73

**Table 3:** Chemical composition of the BOF sludge fine fraction

Elements	Content (%, w/w)
Al	0.31 ± 0.04
As	0.003 ± 0.000
Ba	0.003 ± 0.000
Be	< 0.0001
Bi	< 0.000025
Ca	9.3 ± 0.0
Co	0.0008 ± 0.0000
Cu	0.004 ± 0.001
Cd	< 0.000005
Cr	0.0082 ± 0.0002
Fe	47.8 ± 0.0
K	0.5 ± 0.0
Li	< 0.001
Mg	1.7 ± 0.0
Mn	1.1 ± 0.0
Mo	< 0.0001
Ni	0.002 ± 0.000
P	0.06 ± 0.00
Pb	0.01 ± 0.00
S	0.07 ± 0.00
Sb	0.002 ± 0.000
Sr	< 0.005
Ti	0.025 ± 0.003
V	0.008 ± 0.000
Zn	1.2 ± 0.0
Zr	0.002 ± 0.000

Alkaline elements such as magnesium (1.7 ± 0.0%) and potassium (0.5 ± 0.0%), as well as other metals including zinc (1.2 ± 0.0%) and manganese (1.1 ± 0.0%), were found at lower concentrations in the BOF sludge fine fraction. The zinc content was lower than the values reported by Cantarino et al. (2012) and Kelebek et al. (2004). Nonetheless, the direct reuse of the fine fraction of the BOF sludge in iron or steel production processes would not be indicated, since the zinc content exceeded the maximum recommended value of 0.4% (Smith et al. 2000).

The high iron content was confirmed by the determinations of the total iron and Fe(II) contents, with a total iron content of 45.6 ± 0.5%, consistent with the findings of Cantarino et al. (2012), Matino et al. (2017), and de Oliveira and Bagatini (2019). Of this iron fraction, 41.4 ± 0.5% corresponded to Fe(II), supporting the feasibility of ferrous sulfate production. As reported by Xia et al. (2015), this waste can be considered a valuable iron-bearing resource.

### Production of ferrous sulfate from the BOF sludge fine fraction

In the screening experiments, the factors that presented significant effects (*p* < 0.05) in the production of ferrous sulfate from the BOF sludge fine fraction were the waste amount (*w*), the H_2_SO_4_ solution concentration (*c*), the EtOH volume (*e*), and the leaching time (*t*), as shown by the estimated effects for each factor (Table 4). In contrast to the findings for the coarse sludge fraction, the factor *t* only showed a significant effect when interacting with other factors. This could be explained by the smaller particle size of the fine fraction, which favored iron leaching and shortened the extraction time.

**Table 4:** Effects estimate of the model to optimize the yield of the ferrous sulfate production process from the fine fraction of BOF sludge

Interactions	Effects	*p* value
Average	45.0*	0.0001*
Curvature	29.7*	0.005*
(1) H_2_SO_4_ concentration	− 26.7*	0.001*
(2) Leaching time	− 1.4	0.2
(3) Waste amount	40.9*	0.0004*
(4) Ethanol volume	22.9*	0.001*
1*2	− 1.5	0.2
1*3	1.5	0.2
1*4	− 2.8	0.08
2*3	11.1*	0.006*
2*4	− 12.6*	0.004*
3*4	− 8.2*	0.01*
1*2*3	5.2*	0.03*
1*2*4	2.3	0.1
1*3*4	9.6*	0.008*
2*3*4	9.4*	0.008*

*p < 0.05—level of significance

**Fig. 1:**
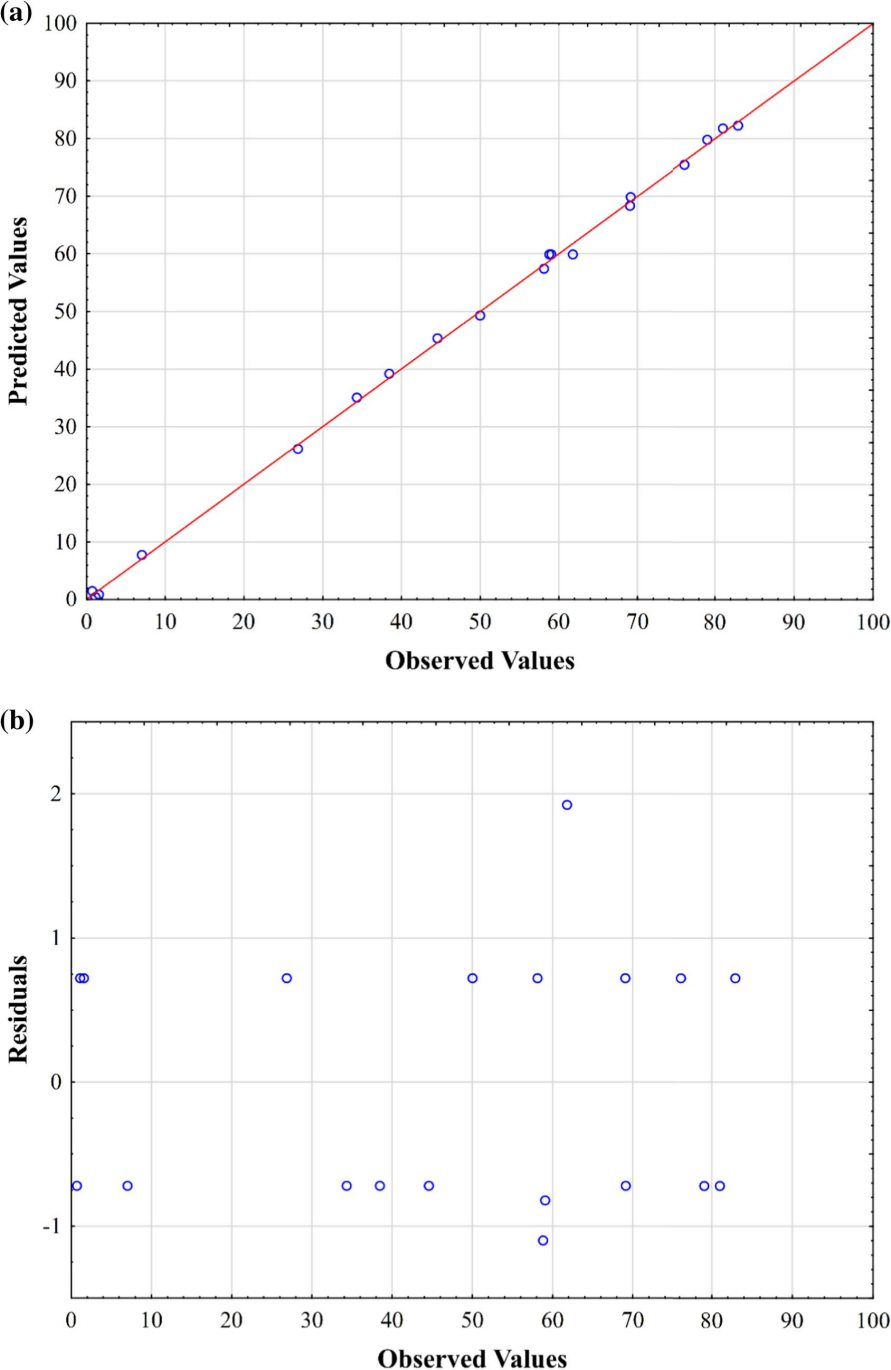
Plots of the linear model from the screening experiments for optimizing ferrous sulfate production: (**a**) observed versus predicted values and (**b**) residual versus observed values

The linear model regression was significant (*p* = 0.0005 < 0.05, *F*_calc_ = 208.1 > *F*_tab_ = 8.7) and the lack of fit was non-significant (*p* = 0.2 > 0.05, *F*_calc_ = 3.0 < *F*_tab_ = 18.51). Although the model presents high *R*^2^ (99.90%) and adjusted *R*^2^ (*R*^2^_adj_ = 99.42%) values (Fig. 1a), the residual degrees of freedom (*df* = 3) were limited, which restricts the statistical foundation for error estimation. This limitation may explain the pattern observed in the residual plot (Fig. 1b), indicating that the assumptions of ANOVA were not fully satisfied. The fact that *R*^2^_adj_ was slightly higher than *R*^2^ suggests that the inclusion of terms improved the model fit beyond what would be expected by chance, rather than indicating overfitting. To ensure that no potentially important effects were overlooked, all variables were subsequently carried forward to DED optimization. Additional data for the screening experiments are available in the Supplementary material (Supplementary Tables 1 and 2).

The DED matrix used to optimize the experimental conditions for the production of ferrous sulfate from the BOF sludge fine fraction is shown in Table 5. The best response, considered the highest yield (81.98%), was obtained in experiment 21, where 8.45 g of FeSO_4_·7H_2_O were produced from 5.00 g of waste. This experiment was performed under the following experimental conditions: 14.5% (v/v) H_2_SO_4_ solution, 110 min leaching time, and 90 mL of EtOH.

**Table 5:** Uncoded and coded (in parentheses) factor values for optimization of ferrous sulfate production from the BOF sludge fine fraction

**Experiment**	***c***^a^ **(%)**	***t***^b^ **(min)**	***w***^c^ **(g)**	***e***^d^ **(mL)**	**FeSO**_**4**_**·7H**_**2**_**O (g)**	**Yield (%)**
01	14.5 (0)	110 (0)	5.01 (0)	120 (1)	7.78	75.36
02	25.0 (0.866)	110 (0)	5.00 (0)	105 (0.5)	5.82	56.44
03	18.0 (0.289)	110 (0)	7.00 (0.817)	105 (0.5)	11.29	78.21
04	18.0 (0.289)	140 (0.791)	5.51 (0.204)	105 (0.5)	8.10	71.32
05	14.5 (0)	110 (0)	5.01 (0)	60 (− 1)	5.10	49.39
06	4.0 (− 0.866)	110 (0)	5.00 (0)	75 (− 0.5)	4.71	45.71
07	11.0 (− 0.289)	110 (0)	3.00 (− 0.817)	75 (− 0.5)	1.22	19.64
08	11.0 (− 0.289)	80 (− 0.791)	4.50 (− 0.204)	75 (− 0.5)	5.88	63.42
09	4.0 (− 0.866)	110 (0)	5.01 (0)	105 (0.5)	6.09	59.03
10	11.0 (− 0.289)	110 (0)	3.00 (− 0.817)	105 (0.5)	1.41	22.81
11	11.0 (− 0.289)	80 (− 0.791)	4.50 (− 0.204)	105 (0.5)	6.41	69.04
12	25.0 (0.866)	110 (0)	5.00 (0)	75 (− 0.5)	7.26	70.46
13	21.5 (0.577)	110 (0)	3.00 (− 0.817)	90 (0)	0.18	2.91
14	21.5 (0.577)	80 (− 0.791)	4.51 (− 0.204)	90 (0)	6.15	66.21
15	18.0 (0.289)	110 (0)	7.00 (0.817)	75 (− 0.5)	6.84	47.41
16	7.5 (− 0.577)	110 (0)	7.00 (0.817)	90 (0)	10.23	70.87
17	14.5 (0)	80 (− 0.791)	6.50 (0.613)	90 (0)	10.77	80.41
18	18.0 (0.289)	140 (0.791)	5.50 (0.204)	75 (− 0.5)	6.84	60.28
19	7.5 (− 0.577)	140 (0.791)	5.50 (0.204)	90 (0)	8.49	74.82
20	14.5 (0)	140 (0.791)	3.50 (− 0.613)	90 (0)	2.06	28.57
21 (CP)	14.5 (0)	110 (0)	5.00 (0)	90 (0)	8.45	81.98
22 (CP)	14.5 (0)	110 (0)	5.00 (0)	90 (0)	8.24	79.98
23 (CP)	14.5 (0)	110 (0)	5.00 (0)	90 (0)	7.94	77.01

CP, center point

^a^H_2_SO_4_ solution concentration (v/v)

^b^Leaching time

^c^Waste amount

^d^Ethanol volume

**Fig. 2:**
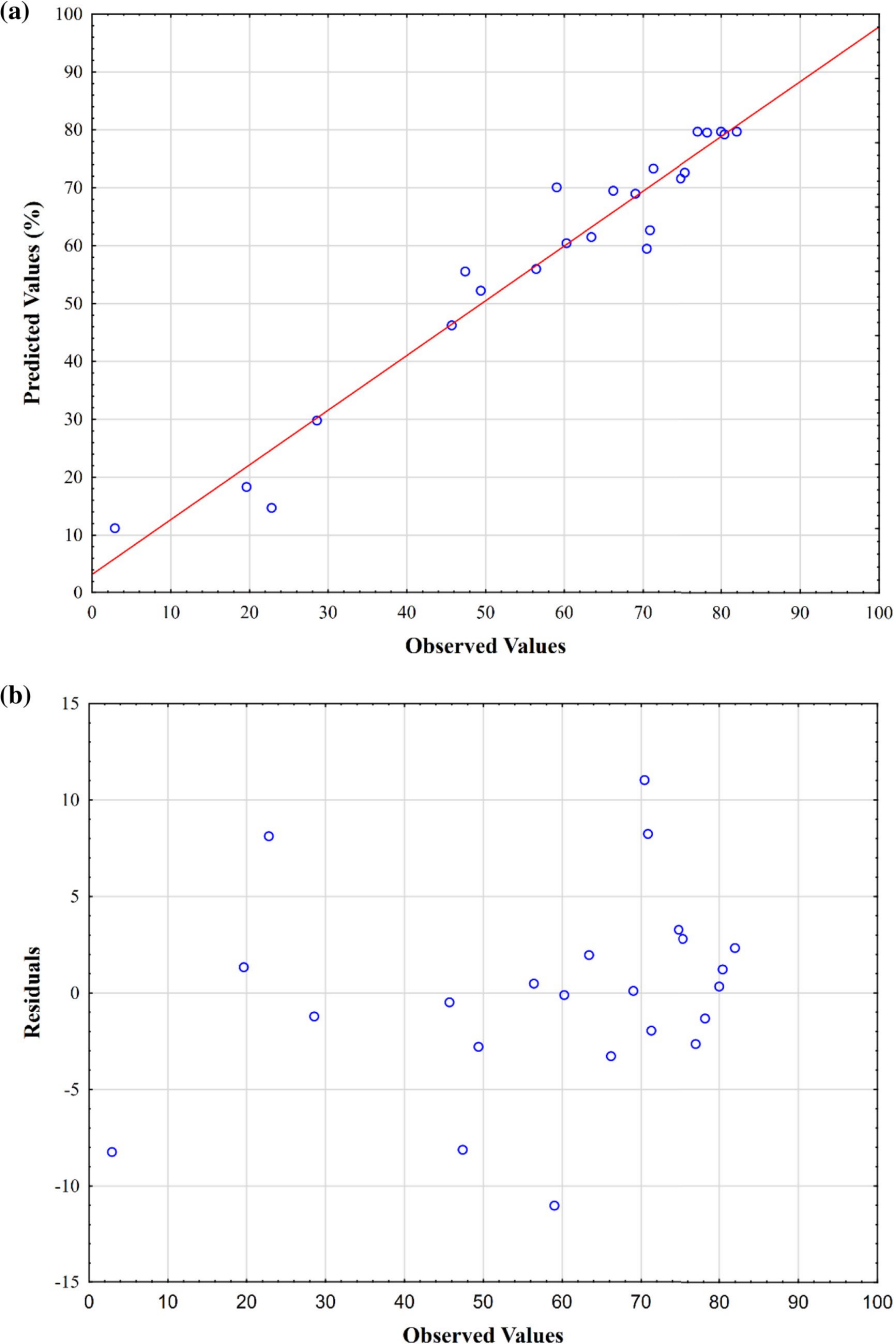
Plots of the quadratic model for ferrous sulfate production optimization: (**a**) observed versus predicted values and (**b**) residual versus observed values

The statistical significance of the quadratic model was evaluated using analysis of variance (ANOVA, Supplementary Table 3). The results showed that regression was statistically significant (*p* = 0.001 < 0.05), with the ratio between the quadratic mean of the regression and the residue (*F*_calc_ = 9.9) being higher than the theoretical *F* distribution (*F*_tab_ = 2.64). The lack of fit was not significant (*p* = 0.06 > 0.05, *F*_calc_ = 15.00 < *F*_tab_), confirming the achievement of a robust regression model. The *R*^2^ (94.56%) and *R*^2^_adj_ (85.04%) values indicate a good fit of the model to the experimental data. The observed versus predicted values plot (Fig. 2(a)) confirmed the adequacy of the model, while the residuals versus observed values plot (Fig. 2(b)) showed a random distribution of errors around zero, with no systematic patterns. Nevertheless, the residual degrees of freedom were limited (*df* = 8), which restricts the statistical foundation for error estimation. This limitation can lead to overfitting, and the decrease from *R*^2^ to *R*^2^_adj_ corroborates this concern. Even so, the diagnostic plots support the assumptions of ANOVA, which were reasonably satisfied. Therefore, while the models offer valuable insights, their interpretation should be approached with caution. Importantly, the Doehlert optimization and experimental validation confirmed the adequacy of the models under the optimized conditions.

The most significant effects in the production of ferrous sulfate from the BOF sludge fine fraction were observed for the waste amount and the H_2_SO_4_ solution concentration. The leaching time had the smallest effect, as expected from the screening experiments. In that stage, leaching time did not appear as a significant factor, likely because its effect was overshadowed by the dominant influence of H_2_SO_4_ concentration, waste amount, and ethanol volume within the wide experimental domain. In contrast, the DED optimization employed narrower ranges for leaching time and acid concentration, which increased the sensitivity of the system to time variations. As a result, leaching time became partly significant in the quadratic model, reflecting its nonlinear behavior and dependence on interactions with other factors. Under milder acid conditions, the duration of leaching becomes more critical to achieving efficient extraction. The estimated effects are shown in Table 6.

**Table 6:** Effects estimate for the quadratic model by DED based on process yield

Factor	Effect	*p* value
Average	79.7*	0.0003*
(1) H_2_SO_4_ concentration (L)^a^	− 0.4	0.8
H_2_SO_4_ concentration (Q)^b^	− 34.9*	0.01*
(2) Leaching time (L)^a^	− 11.0*	0.03*
Leaching time (Q)^b^	− 8.4	0.08
(3) Waste amount (L)^a^	51.2*	0.001*
Waste amount (Q)^b^	− 65.2*	0.002*
(4) Ethanol volume (L)^a^	20.4*	0.01*
Ethanol volume (Q)^b^	− 34.6*	0.02*
1*2	− 13.8	0.1
1*3	7.3	0.3
1*4	− 27.3*	0.03*
2*3	7.0	0.3
2*4	5.4	0.4
3*4	36.7	0.02

*p < 0.05—level of significance

^a^L = linear terms

^b^Q = quadratic terms

The second-order polynomial model describing the ferrous sulfate production is shown in Eq. (1). This model considered all the factors and interactions that had a significant effect and/or a significant regression coefficient (*p* < 0.05).

1 $$Yield = -23.53 + 10.01c- 0.16{(c)}^{2} + 57.84w- 8.15({w)}^{2} + 2.57e- 0.02({e)}^{2}- 0.04c\times e + 0.31w\times e$$

**Fig. 3:**
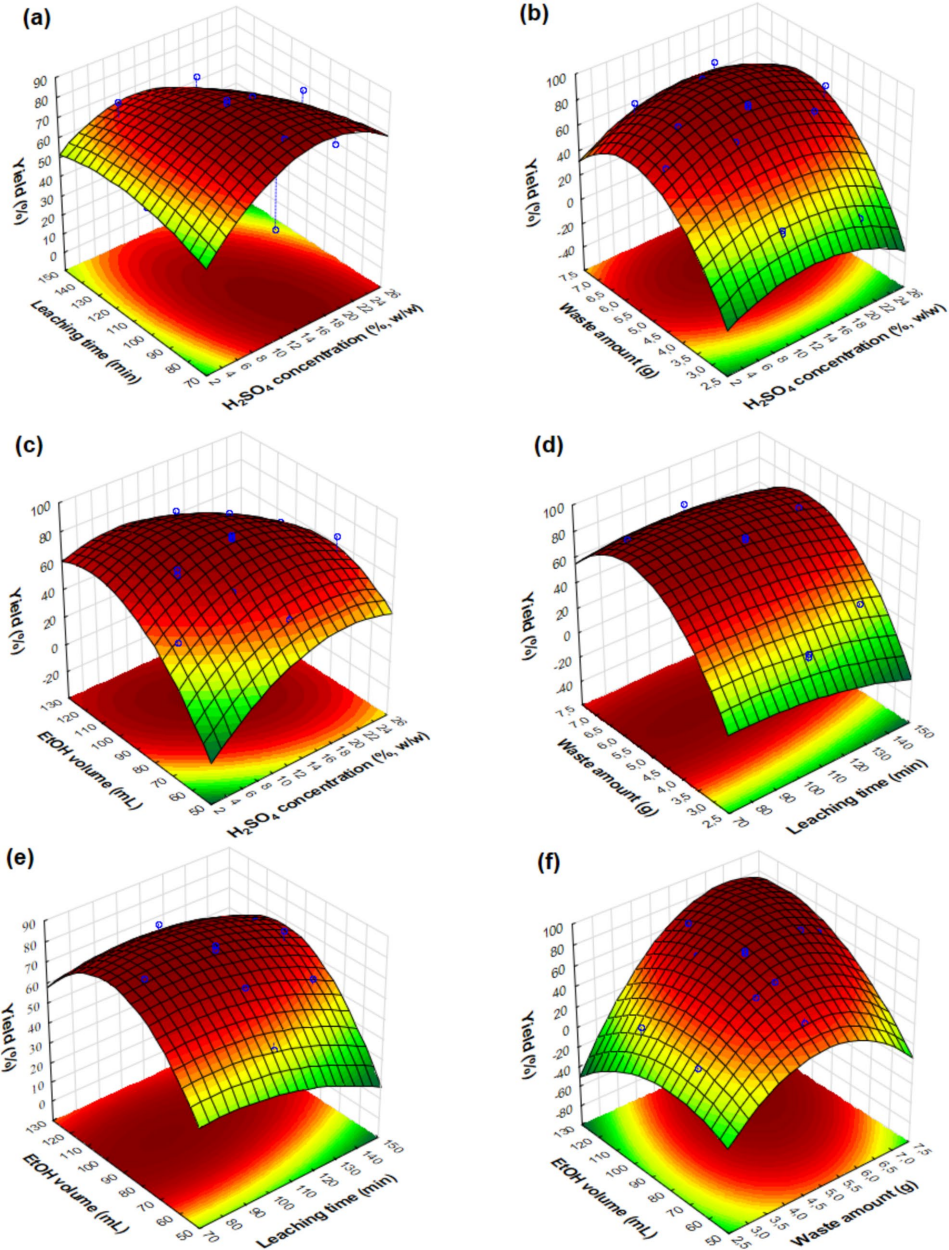
Response surfaces based on the DED matrix experiments for the ferrous sulfate yield as a function of (**a**) leaching time and H_2_SO_4_ concentration, (**b**) waste amount and H_2_SO_4_ concentration, (**c**) EtOH volume and H_2_SO_4_ concentration, (**d**) waste amount and leaching time, (**e**) EtOH volume and leaching time, and (**f**) EtOH volume and waste amount. All the factors were fixed at the center points

Response surfaces for the yield of ferrous sulfate, based on Eq. (1), are shown in Fig. 3(a)–(f), indicating the experimental domain for maximum ferrous sulfate production. In contrast to the leaching process, the increase in the mass of waste improved the ferrous sulfate yield, as also reported in the first part of this study (Maia et al. 2020).

**Fig. 4:**
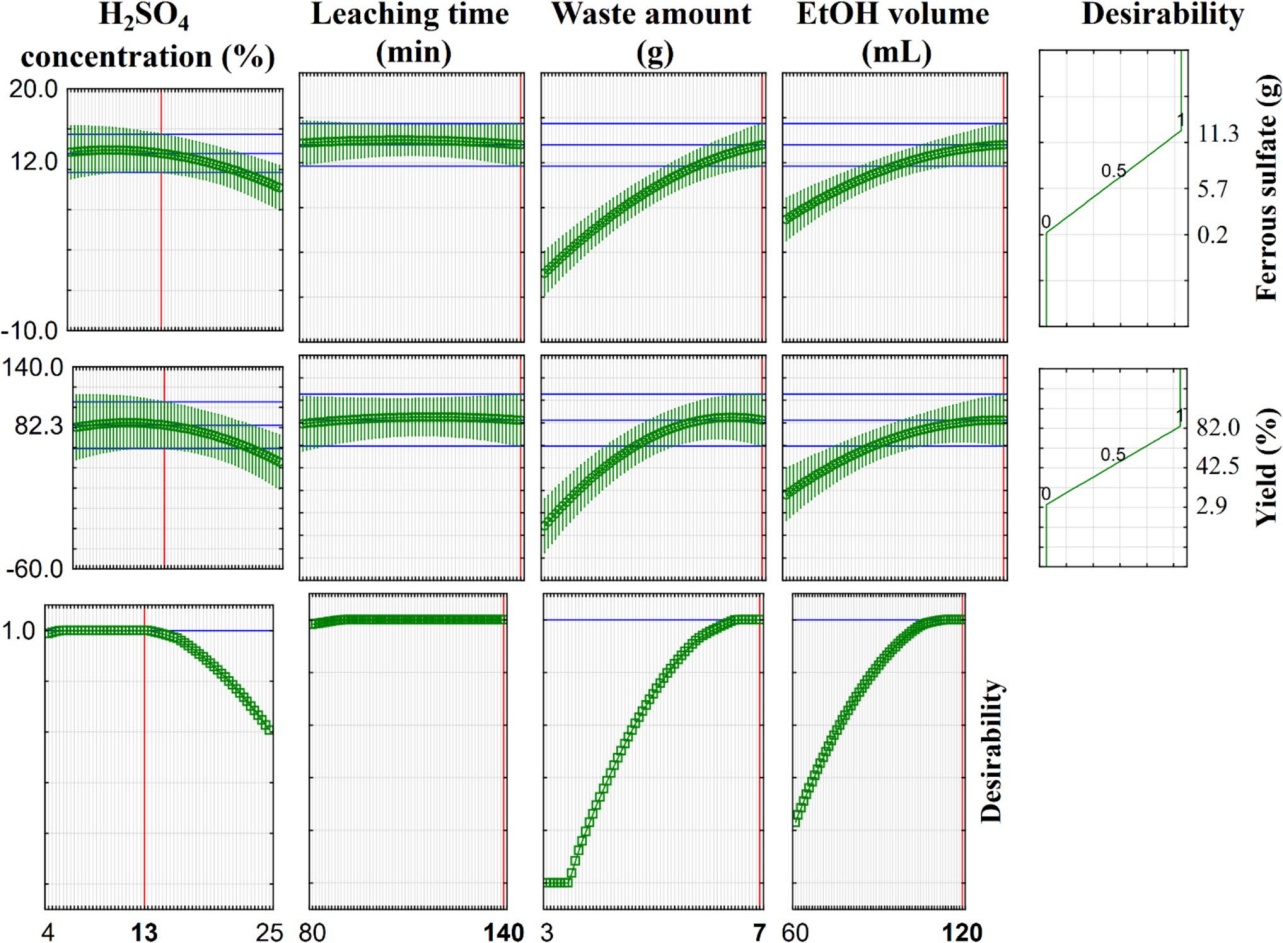
Profiles of predicted values and desirability for optimization of ferrous sulfate production from the fine fraction of the BOF sludge

A desirability function was employed for simultaneous optimization of multiple responses (mass of ferrous sulfate heptahydrate and process yield), as shown in Fig. 4. The optimum condition indicated by the desirability function was using 7.00 g of waste (solid-to-liquid ratio (*SLR*) of 0.14 g mL^−1^), 13% (v/v) H_2_SO_4_ solution, a leaching time of 140 min, and 120 mL of EtOH. The predicted maximum response values were 12.0 g of ferrous sulfate heptahydrate and 82.3% yield. The optimum condition was validated, obtaining 13.1 ± 0.2 g of ferrous sulfate heptahydrate and a yield of 91 ± 1%, where the maximized responses were due to the higher levels for most of the factors (leaching time, EtOH volume, and waste amount).

The fine BOF sludge fraction required a shorter leaching time (140 min) than the coarse fraction (200 min) (Maia et al. 2020), which could be explained by the smaller particle size of the fine fraction and its composition (mostly FeO), as reported by Cantarino et al. (2012). These results demonstrated the efficiency of the processes developed and optimized for the recovery of iron from the coarse and fine BOF sludge fractions. As discussed by Siedlecka (2020), a shorter leaching time reduces production costs, increasing the economic feasibility of the process. Although an increase in the sulfuric acid solution concentration improved the leaching of iron, the use of more concentrated sulfuric acid solutions impaired the crystallization of ferrous sulfate. According to Trung et al. (2011), Fe(II) ions can be oxidized to Fe(III) in more acidic solutions with pH ~ 0.5. In addition, a yellowish color was observed in the iron-rich leachate solution, which could have been related to the presence of Fe(III) in solution.

Following optimization of ferrous sulfate production, the chemical composition of the leachate obtained based on the desirability function (Fig. 4) was determined, as shown in Table 7. The largest fraction of iron was recovered using a low acid concentration and a short leaching time, at 25.0 ± 0.1 °C. The leachate showed high contents of iron (32.0 ± 0.5% w/w) and sulfur (9.6 ± 0.2% w/w). Other elements, including manganese, zinc, and magnesium, showed leaching percentages above 60%. The lack of selectivity in zinc and iron extraction during acid leaching was also reported by Trung et al. (2011), Kelebek et al. (2004), and Siedlecka (2020).

**Table 7:** Chemical composition of the leachate obtained from the BOF sludge fine fraction under the optimum conditions determined by the desirability function (7.00 g of waste, 13% (v/v) H_2_SO_4_ solution, and leaching time of 140 min)

Element	Content (% w/w)
Al	0.14 ± 0.02
As	< 0.000003
Ba	0.0005 ± 0.0000
Be	< 0.000006
Bi	< 0.000006
Ca	0.23 ± 0.03
Co	0.0004 ± 0.0001
Cu	0.002 ± 0.000
Cd	< 0.0000003
Cr	0.004 ± 0.001
Fe	32.0 ± 0.5
K	0.2 ± 0.1
Li	0.0003 ± 0.001
Mg	1.0 ± 0.1
Mn	0.71 ± 0.07
Mo	< 0.000006
Ni	0.0009 ± 0.000
P	0.05 ± 0.02
Pb	0.004 ± 0.001
S	9.6 ± 0.2
Sb	0.0004 ± 0.0002
Sr	< 0.000006
Ti	0.009 ± 0.002
V	0.004 ± 0.001
Zn	0.75 ± 0.05
Zr	0.001 ± 0.000

The iron recovery observed in the present study was higher than that reported by Siedlecka (2020), who investigated the acid leaching of BOF sludge using solutions containing 2.5 mol L^−1^ H_2_SO_4_ (13.3% v/v) and 1 mol L^−1^ HCl. The authors reported a leaching efficiency of 33% under the following experimental conditions: leaching time of 120 min, temperature of 60 °C, stirring speed of 500 rpm, and *SLR* of 0.1 g mL^−1^.

As discussed by Maia et al. (2020), the proposed ferrous sulfate production process could be improved by recirculating the residual solution corresponding to the non-precipitated ferrous sulfate fraction. For recovery, the residual solution should be subjected to distillation to separate and recover EtOH, which could also be reused after membrane separation, as proposed by Kesieme et al. (2013) and Kesieme and Aral (2015). After distillation, both the leaching agent (H_2_SO_4_ solution) and the EtOH could be reused, returning to the process and reducing production costs.

### Characterization of the ferrous sulfate crystals

**Fig. 5:**
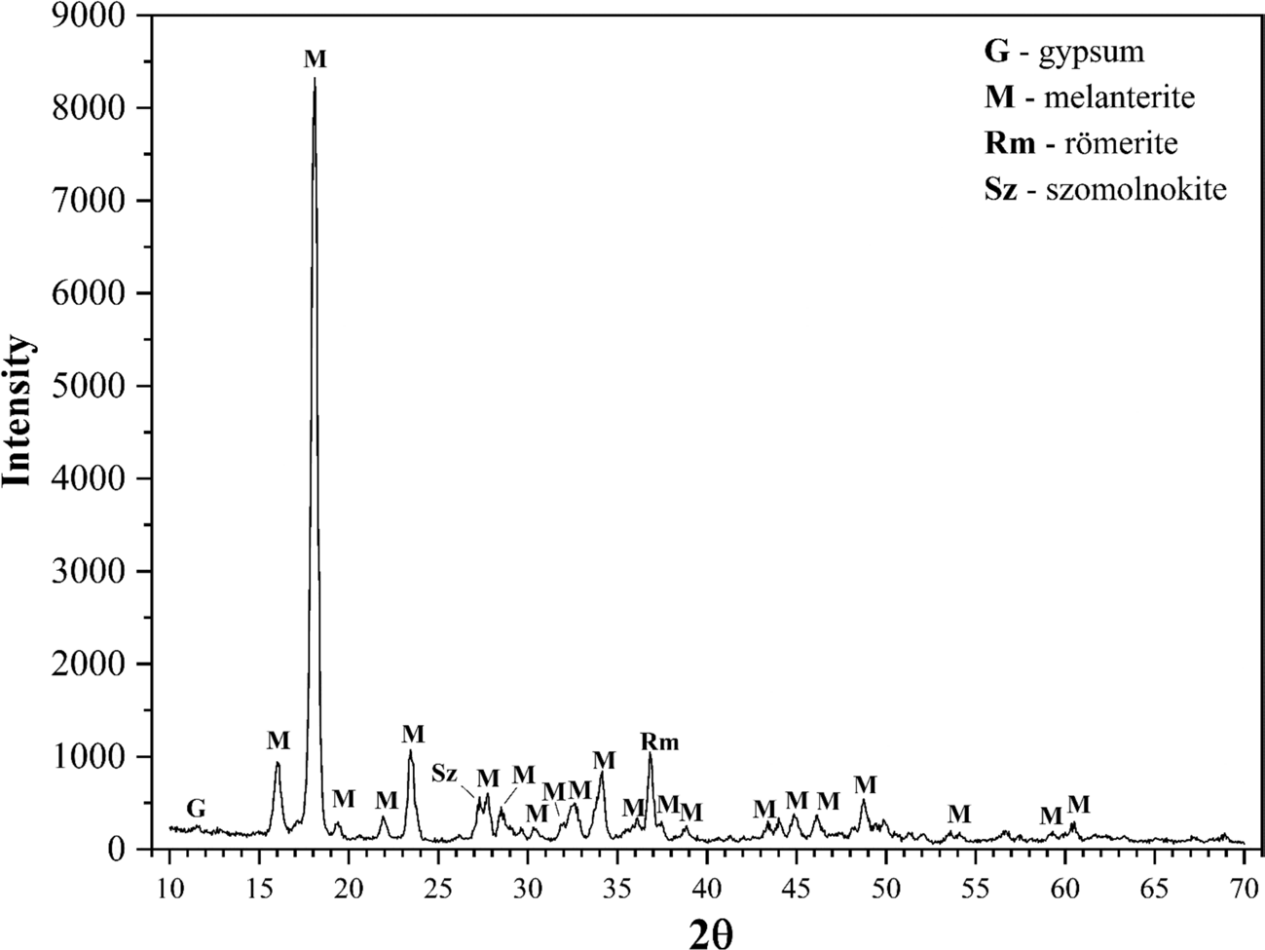
X-ray diffractogram of the ferrous sulfate obtained from the fine fraction of the BOF sludge. Experimental conditions: H_2_SO_4_ concentration of 13% (v/v), leaching time of 140 min, 7.00 g of waste, and 120 mL of EtOH

X-ray diffraction analysis indicated that the ferrous sulfate produced from the BOF sludge fine fraction was mainly composed of melanterite (FeSO_4_·7H_2_O), as shown in Fig. 5. The main diffraction signal at 2θ of 18.1° corresponded to melanterite, confirming the successful crystallization of ferrous sulfate heptahydrate. Other phases identified were szomolnokite (FeSO_4_·H_2_O), römerite (Fe^2+^Fe^3+^_2_(SO_4_)_4_·14H_2_O), and gypsum (CaSO_4_·2H_2_O), as confirmed by scanning electron microscopy (SEM) coupled to energy dispersive X-ray spectroscopy (EDX).

**Fig. 6:**
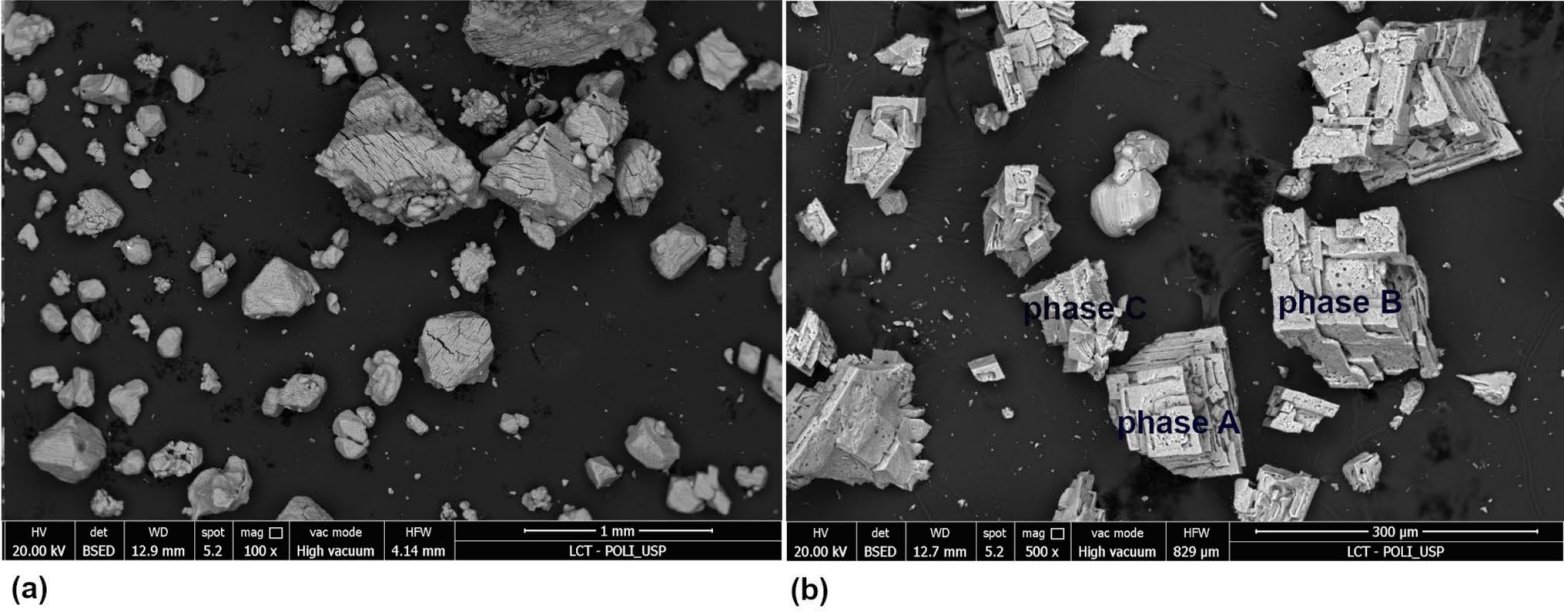
SEM images of (**a**) analytical grade ferrous sulfate (at × 100 magnification) and (**b**) the ferrous sulfate produced from the BOF sludge fine fraction (at × 500 magnification) (conditions: 13% (v/v) H_2_SO_4_ solution, leaching time of 140 min, waste amount of 7.00 g, and 120 mL of EtOH)

Figure 6(a)-(b) shows SEM micrographs of analytical grade ferrous sulfate heptahydrate (at × 100 magnification) and the ferrous sulfate produced from the BOF sludge fine fraction (at × 500 magnification). Smaller ferrous sulfate particles were obtained from the sludge (Fig. 6(b)), with shapes close to those of the particles in the analytical ferrous sulfate sample (Fig. 6(a)).

**Fig. 7:**
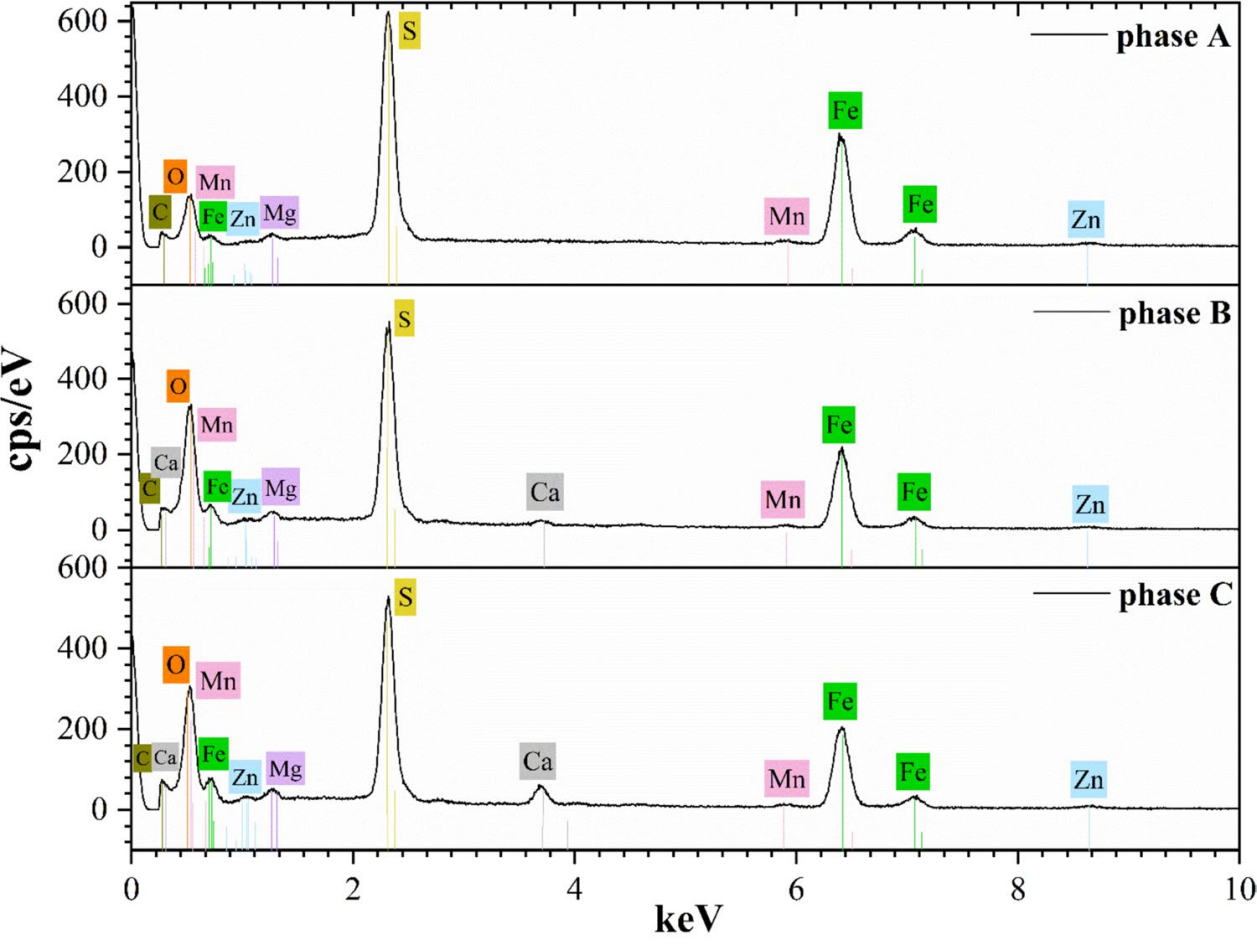
SEM–EDX analysis of the ferrous sulfate obtained using 13% (v/v) H_2_SO_4_ solution, leaching time of 140 min, waste amount of 7.00 g, and 120 mL of EtOH

The EDX spectra revealed the predominance of iron and sulfur, confirming the formation of ferrous sulfate (Fig. 7). Other elements (Mn, Mg, Ca, and Zn) were also identified in phases A, B, and C of the ferrous sulfate samples (Fig. 7), as expected considering the sludge composition. The presence of calcium was related to gypsum crystals (CaSO_4_·2H_2_O), which were identified by XRD analysis, as also reported by Vigânico et al. (2011). The presence of magnesium, manganese, and zinc could be attributed to the composition of the waste used in the production of the ferrous sulfate. As discussed by Maia et al. (2020), the presence of these elements would not compromise the application of the ferrous sulfate as an intermediate in the production of ferric coagulant.

**Fig. 8:**
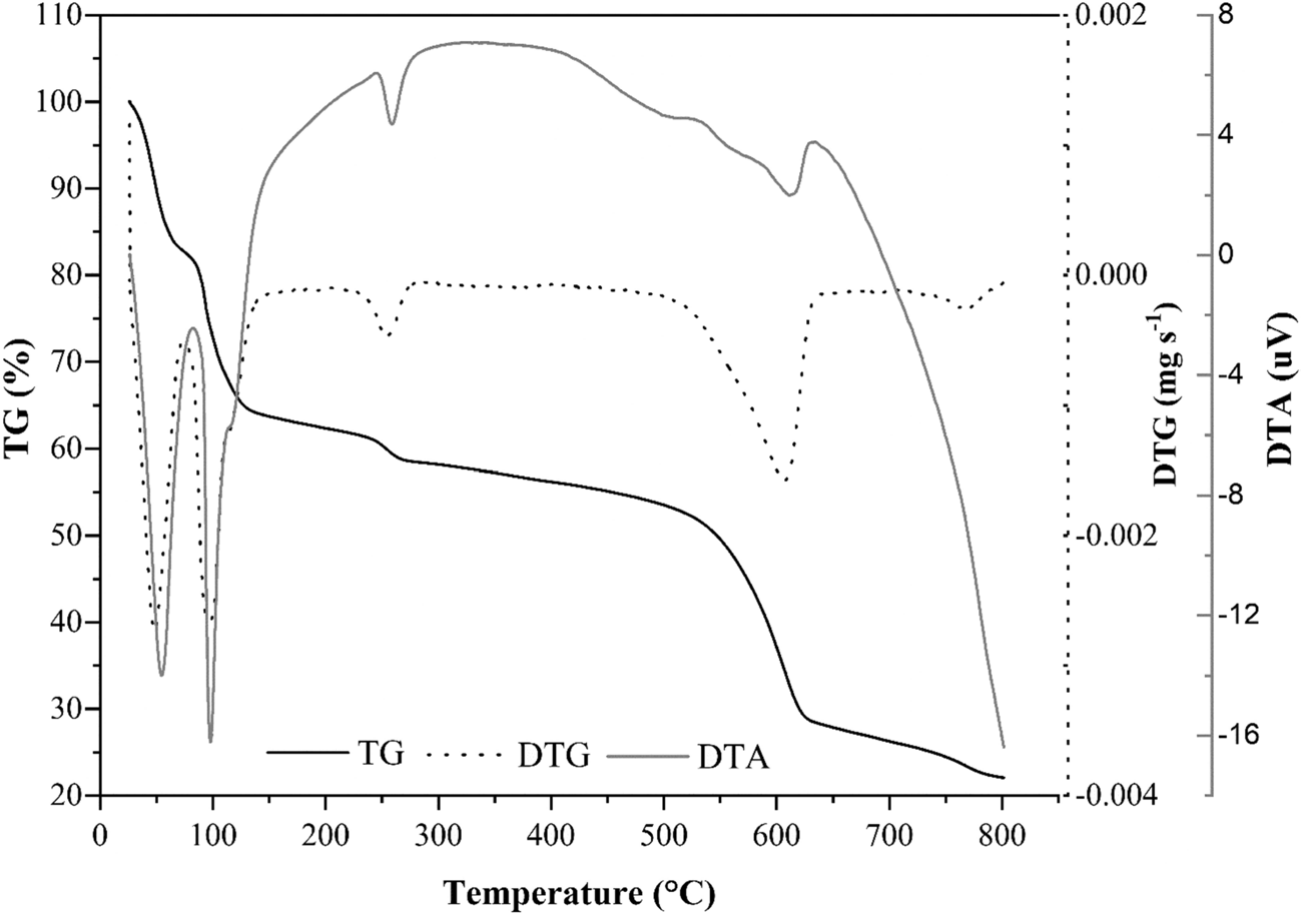
Thermogravimetry curves for decomposition of the ferrous sulfate heptahydrate sample obtained using H_2_SO_4_ at a concentration of 13% (v/v), leaching time of 140 min, 7.00 g of waste, and 120 mL of EtOH. TG, thermogravimetric curve (mass loss, %); DTG, differential thermogravimetric curve (mass loss rate, mg s^−1^); DTA, differential thermal analysis curve (µV)

The decomposition curve of the ferrous sulfate produced from the BOF sludge fine fraction (conditions: 7.00 g of waste, 13% (v/v) H_2_SO_4_ solution, leaching time of 140 min, and 120 mL of EtOH) is shown in Fig. 8. The initial events corresponded to the removal of sample moisture and hydration molecules of ferrous sulfate. The events at 48, 98, and 253.6 °C, clearly shown in the differential thermogravimetric (DTG) curve, were related to the dehydration of ferrous sulfate heptahydrate and corresponded to weight losses of 17.1, 19.0, and 5.3%, respectively. Similarly, Yani and Zhang (2010) reported a significant weight loss between 30 and 140 °C, attributed to the loss of water from ferrous sulfate heptahydrate. The differential thermal analysis (DTA) showed endothermic events at 55 and 98 °C, associated with dehydration of the ferrous sulfate heptahydrate (loss of six water molecules), as also reported by Siedlecka (2020). The endothermic event at 259 °C (weight loss of 5.3%) corresponded to the loss of the seventh water molecule. The fourth endothermic event (at 612 °C) was associated with decomposition of the sulfate (weight loss of 30.2%), in agreement with Yani and Zhang (2010), who reported that such desulfurization reactions occur between 540 and 680 °C. Additional data for the thermogravimetric analysis are available in Supplementary Table 4.

The presence of other events in the thermogravimetric analysis suggested impurities in the sample, which could have been related to the elements identified by SEM–EDX analysis, including calcium, magnesium, manganese, and zinc. The suitability of ferrous sulfate as an intermediate product to obtain ferric coagulant depends on the concentrations of these impurities in the material. For the final product (coagulant), an oxidation step is required, producing the ferric sulfate (Fe_2_(SO_4_)_3_) commonly employed as a coagulant in most WTPs.

### Production of ferric coagulants from the coarse and fine fractions of BOF sludge

An efficient oxidation process was achieved using the FeSO_4_·7H_2_O:H_2_O_2_ ratio of 1:0.4 (w/v), resulting in oxidation efficiencies of 91.549 ± 0.005% for FC-I (from the BOF sludge coarse fraction) and 93.08 ± 0.08% for FC-II (from the BOF sludge fine fraction). Therefore, this ratio was defined as the best condition for coagulant production. The Fe(III) contents of FC-I and FC-II were 19.45 and 18.90%, respectively. These results showed that the coagulants produced from the coarse and fine fractions of the BOF sludge were able to meet the specifications required for commercialization in Brazil (iron content of at least 12% w/w) (Menezes et al. 2017).

Table 8 shows the chemical compositions of the coagulants produced from the coarse and fine BOF sludge fractions by acid leaching. The FC-I and FC-II coagulants had high total iron contents of 21.25 and 20.30%, respectively, with low impurity levels. According to European standard BS EN 890:2023 (CEN 2023), the product typically contains not less than a mass fraction of 30% iron(III) sulfate (Fe_2_(SO_4_)_3_)—which corresponds to approximately 8.4% of total iron—and the standard explicitly applies to solutions with varying iron contents. Therefore, both FC-I and FC-II far exceed the typical commercial baseline for iron concentration defined in the standard. The European standard establishes purity criteria based on concentration limits of the chemical parameters specified in milligrams per kilogram of Fe(III) (CEN 2023). As shown in Table 8, FC-I and FC-II contained concentrations of As, Cd, Cr, Ni, Pb, and Sb below the limits established in the BS EN 890:2023 standard for coagulant Type III (CEN 2023), indicating a low level of impurities. These results demonstrated that the crystallization of ferrous sulfate using EtOH led to a product with a high degree of purity, as also reported by Siedlecka (2020).

**Table 8:** Chemical characterization of coagulants FC-I and FC-II, obtained from coarse and fine BOF sludge fractions, compared with maximum permitted element levels according to European standard BS EN 890:2023

Element	FC-I (mg kg^−1^)	Limit FC-I (mg kg^−1^)^a^	FC-II (mg kg^−1^)	Limit FC-II (mg kg^−1^)^a^
Al	6.29	–	7.51	–
As	< 0.01	9.73	< 0.01	9.43
Ba	< 0.25	–	< 0.25	–
Be	< 0.02	–	< 0.02	–
Bi	< 0.02	–	< 0.02	–
Ca	773.17	–	1723.49	–
Co	6.66	–	6.04	–
Cu	< 0.01	–	14.29	–
Cd	< 0.001	9.73	< 0.001	9.43
Cr	< 0.02	97.26	< 0.02	94.26
Fe	212,467.55	–	202,531.72	–
K	< 0.02	–	30.92	–
Mg	2231.96	–	6492.35	–
Mn	1891.61	–	3102.04	–
Mo	< 0.02	–	< 0.02	–
Ni	18.62	97.26	11.83	94.26
Li	< 0.25	–	< 0.25	–
P	19.61	–	< 0.02	–
Pb	21.21	77.81	18.48	75.41
S	97,231.96	–	96,739.70	–
Sb	3.45	11.67	2.20	11.31
Sr	< 0.02	–	< 0.02	–
Ti	< 0.01	–	7.76	–
V	10.85	–	11.21	–
Zn	618.41	–	5740.86	–
Zr	7.03	–	7.39	–

^a ^The concentration limits were determined in milligrams per kilogram of Fe(III), i.e., they were calculated by multiplying the concentration limit of each contaminant by the Fe(III) content in the coagulant (type III) (CEN 2023)

### Performance of the ferric coagulants in water clarification

**Fig. 9:**
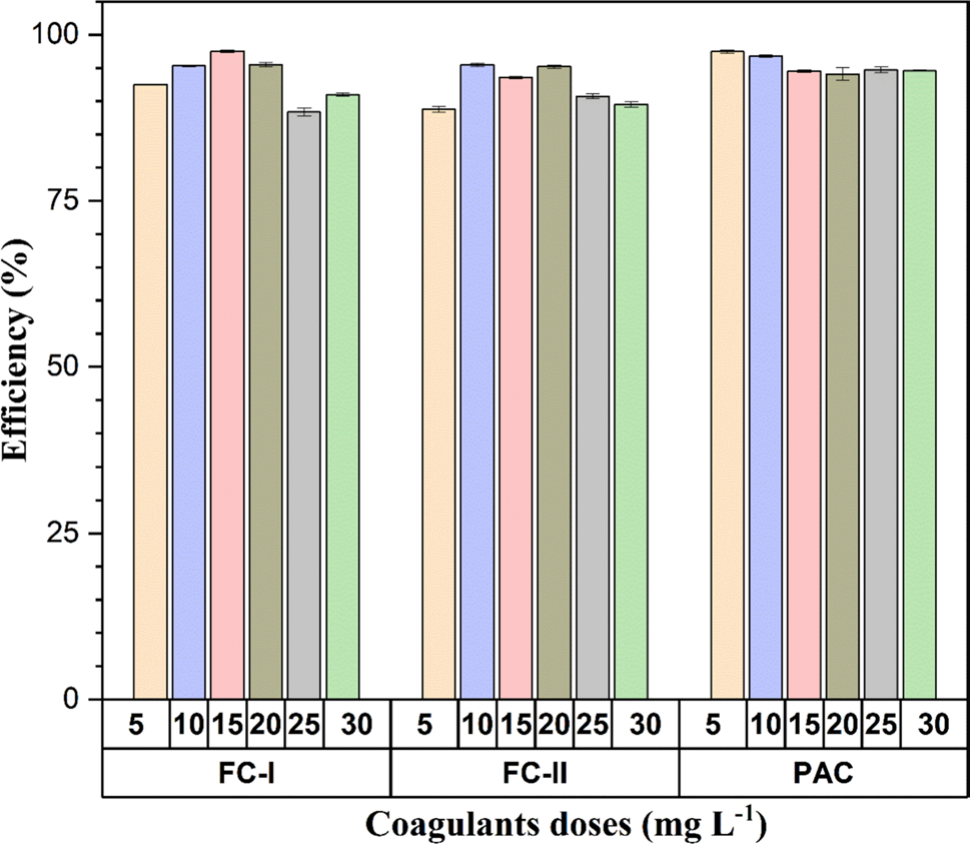
Optimization experiments for clarification of high-turbidity water using different coagulants

Jar tests (5–30 mg Fe(III) L^−1^ at pH 6.2–6.9 for ferric-based coagulants; 5–30 mg Al(III) L^−1^ at pH 7.5–7.9 for PAC) were conducted to determine the optimal conditions for turbidity removal. The evaluation included coagulants FC-I and FC-II, produced from the coarse and fine fractions of BOF sludge, respectively, as well as a commercial coagulant. Results of the experiments carried out to establish the optimal dose for each coagulant are shown in Fig. 9, while comprehensive data from these tests are provided in Supplementary Tables 5, 6, and 7. For FC-I, the optimal condition was achieved at a coagulant dose of 15 mg Fe(III) L^−1^ at a pH of 6.6, resulting in a turbidity removal efficiency of 95 ± 1% (Table 9). In contrast, FC-II coagulant required a lower coagulant dose of 10 mg Fe(III) L^−1^ at pH 6.7, yielding 94.1 ± 0.9% removal of suspended particles (Table 9). PAC exhibited the highest performance at the lowest coagulant dose (5 mg Al(III) L^−1^) at pH 7.55, achieving a turbidity removal efficiency of 97 ± 1%.

**Table 9:** Optimized conditions for turbidity removal from natural water using ferric coagulants FC-I and FC-II derived from BOF sludge, in comparison with a commercial coagulant

Parameter	FC-I	FC-II	PAC
Coagulant dose (mg L^−1^)	15	10	5
Coagulation pH	6.6 ± 0.0	6.7 ± 0.2	7.55 ± 0.1
Flocculation time (min)	20	20	20
Settling velocity (cm min^−1^)	1.74	1.74	1.74
Remaining turbidity (NTU)	10 ± 2	12 ± 2	6 ± 1
Removal efficiency (%)	95 ± 1%	94.1 ± 0.9%	97 ± 1%

There is no standard regarding the remaining turbidity after the coagulation, flocculation, and sedimentation steps of water treatment. The drinking water guidelines only establish a value for the turbidity after the filtration step. European Union Directive 2020/2184 (European Parliament and Council 2020) and the National Primary Drinking Water Regulations of the Environmental Protection Agency (EPA 2025) recommend turbidity below 0.3 NTU in 95% of water samples after filtration. This value was established to avoid the formation of trihalomethanes during the water disinfection step. The residual turbidity after coagulation (10 ± 2 NTU for FC-I, 12 ± 2 NTU for FC-II, and 6 ± 1 NTU for PAC) can be effectively removed during the subsequent filtration step, thereby ensuring compliance with drinking water standards.

The ferric coagulants derived from BOF sludge (FC-I and FC-II) achieved turbidity removals of 95 ± 1% and 94.1 ± 0.9%, respectively, values comparable to those obtained with commercial PAC under similar conditions (Table 9) and were also more efficient than alternative coagulants, such as *Moringa oleifera* (dos Santos et al. 2016a, b). Statistical analysis using the *t* test (*p* < 0.05) confirmed that the performance of FC-I was not significantly different from that of the commercial coagulant (*p* = 0.06 > 0.05), whereas FC-II exhibited significantly lower efficiency compared to PAC (*p* = 0.001 < 0.05). The study advances beyond earlier approaches by demonstrating that BOF sludge, a steelmaking residue with challenging management, can be transformed into competitive coagulants. Similar valorization strategies have been reported for acid mine drainage, where selective precipitation enables the production of Fe_2_(SO_4_)_3_ with effective performance in water treatment (Oré Núñez et al. 2019), reinforcing the broader potential of waste-to-resource strategies. Overall, the removal efficiency values indicated that both coagulants (FC-I and FC-II) have potential for application in water treatment. It should be noted, however, that the operational parameters evaluated in this study (pH 6.2–6.9 and coagulant dose of 5–30 mg Fe^3+^ L^−1^) were relatively narrow and based on a single high-turbidity surface water matrix. Performance may vary under different water qualities, such as lower turbidity, distinct alkalinity, or higher natural organic matter content. Future work must further evaluate these aspects to expand the application of alternative coagulants.

### Cost estimation for the production of ferrous sulfate and ferric coagulant from the fine and coarse fractions of BOF sludge

As described by Turton et al. (2008), the final price of a product manufactured on an industrial scale depends on a variety of factors, including the following: (i) direct manufacturing costs (raw materials and labor); (ii) fixed manufacturing costs (taxes, insurance, and depreciation); and (iii) general costs (management, sales, financing, and research functions). In the case of emerging technologies, preliminary economic analysis is particularly important to enable assessment of the potential for scaling up from bench experiments, identifying hotspots for technological improvements (van der Spek et al. 2017).

**Fig. 10:**
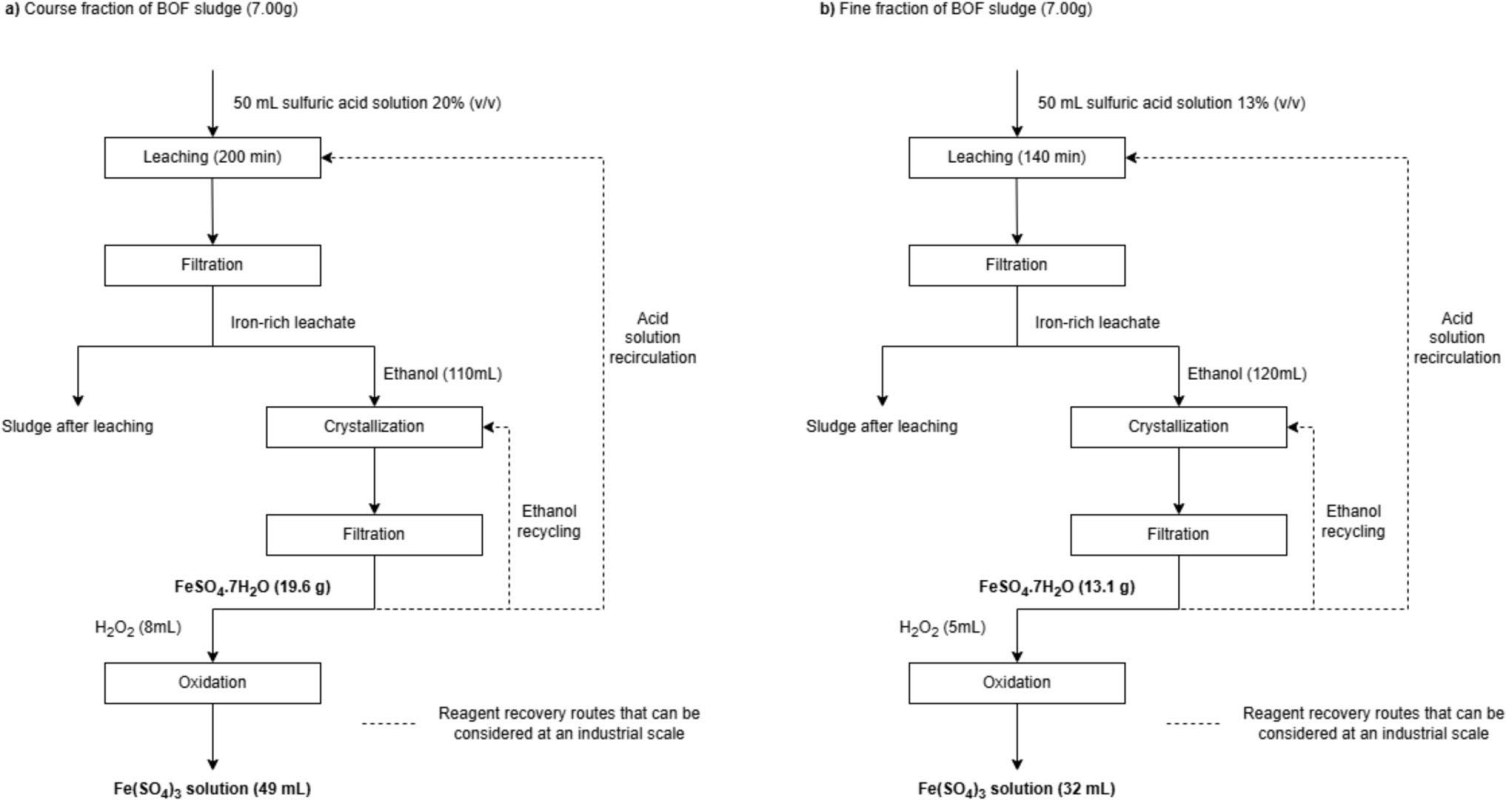
Flowsheets for the production of ferrous sulfate and ferric sulfate from the coarse (**a**) and fine (**b**) fractions of BOF sludge

Schematic flowsheets of the processes for recycling the coarse and fine fractions of BOF sludge are shown in Fig. 10. Based on the process mass balance for the coarse fraction, the estimated production costs for ferrous sulfate (FS-I) and ferric coagulant (FC-I) were 7.4 and 2.8 US$ kg^−1^, respectively (see Supplementary Table 13). For the fine fraction, the values were US$ 10.2 kg^−1^ (for FS-II) and US$ 3.7 kg^−1^ (for FC-II) (see Supplementary Table 13). These values are indicative of bench-scale conditions only, with substantial uncertainties when extrapolated to industrial scale.

Therefore, a simple sensitivity analysis was conducted to evaluate the impact of H_2_SO_4_ and EtOH recovery rates on production costs of the ferric coagulants (FC-I and FC-II), as detailed in Table 10. The analysis varied recovery rates from 0 to 95%, demonstrating the critical influence of reagent recovery efficiency on process economics.

**Table 10:** Sensitivity of production costs (US$ kg^−1^ coagulant) to reagent recovery rates

Recovery rate (%)	Coarse fraction (FC-I)	Fine fraction (FC-II)
0	9.43	13.96
20	7.77	11.40
40	6.11	8.84
60	4.44	6.28
**80 (base case)**	**2.78**	**3.72**
95	1.53	1.80

The sensitivity analysis (Table 10) demonstrates that production costs are highly sensitive to these assumptions: at 0% recovery, costs increase dramatically to US$ 9.43 kg^−1^ (coarse) and US$ 13.96 kg^−1^ (fine), while 95% recovery reduces costs to 1.53–1.80 US$ kg^−1^. These results confirm that reagent recovery efficiency is a critical economic parameter that requires validation at a larger scale.

Although the costs of producing FS-II and FC-II (from the fine fraction of BOF sludge) were higher than for FS-I and FC-I (from the coarse fraction of BOF sludge), it should be noted that the management of the fine sludge fraction is more technologically challenging, due to the lower levels of iron and higher levels of zinc in this fraction (Cantarino et al. 2012; Kelebek et al. 2004). Additional data for the cost estimation are available in the Supplementary Material (Supplementary Tables 8, 9, 10, 11, 12, and 13).

Even using a lower concentration of H_2_SO_4_ (13% v/v) and a shorter leaching time (140 min) for the fine sludge fraction, the costs were higher than for the products derived from the coarse sludge fraction. This was due to the lower yield of the process, in terms of the ratio of FeSO_4_·7H_2_O per gram of BOF sludge fine fraction (1.9 g of FeSO_4_·7H_2_O per gram sludge fine fraction), when compared to the coarse sludge fraction (2.8 g of FeSO_4_·7H_2_O per gram sludge coarse fraction). In addition, the ferrous sulfate produced from the fine fraction required a higher volume of ethanol (120 mL) for the precipitation of ferrous sulfate crystals, increasing the cost of obtaining the final product.

For both residues (the coarse and fine fractions of the BOF sludge), the unit operation that most contributed to the final costs of the ferrous sulfate and the ferric coagulant was the crystallization of ferrous sulfate (necessary for purification of the final products). This information can provide useful guidance for developing a scaled-up process. Further in-depth studies should focus on reducing the costs of the proposed technology, aiming to identify alternative strategies for the precipitation of ferrous sulfate, such as the use of lower temperatures (Shaikh et al. 2013).

In 2019, Brazilian crude steel production was 32.5 × 10^6^ tons (Instituto Aço Brasil 2020). According to Cantarino et al. (2012), a Brazilian steelmaking plant generates an average of 6.4 and 19.9 kg of the fine and coarse fractions of BOF sludge, respectively, per ton of steel. Therefore, considering the yields reported here and in the first part of this study (Maia et al. 2020), the potential production of ferrous sulfate from the coarse and fine sludge fractions would be 582.4 × 10^6^ and 1209.4 × 10^6^ tons, respectively. Consequently, the production of ferric coagulant (final product) from the coarse and fine BOF sludge fractions would be 1435.2 × 10^6^ and 2975.1 × 10^6^ tons, respectively.

## Conclusion

The application of steel production waste as raw material to produce ferric coagulants was shown to be feasible, with the potential for recycling the coarse and fine fractions of BOF sludge. The highest yield of ferrous sulfate (91 ± 1%) was obtained for the fine sludge fraction, requiring a shorter leaching time (140 min) than the coarse fraction (200 min). The process efficiencies for oxidation of the ferrous sulfate produced from the coarse and fine sludge fractions were 91.549 ± 0.005% (FC-I) and 93.08 ± 0.08% (FC-II), respectively. The FC-I coagulant had a higher iron content (21.25%) than the FC-II coagulant (20.30%), although both coagulants complied with commercial specifications. Both FC-I and FC-II presented high efficiency in the removal of turbidity from water (95 ± 1 and 94.1 ± 0.9%, respectively), showing that effective coagulants could be produced from steel production wastes. The preliminary bench-scale cost analysis, which considered only reagents, water, and electricity, provided an initial indication that the proposed technology could be economically promising, pending further scale-up assessment. The estimated costs for production of ferric coagulants from the coarse and fine BOF sludge fractions, at a bench scale (7 g waste per batch), were 2.8 and 3.7 US$ kg^−1^, respectively. These values are indicative only, with substantial uncertainties when extrapolated to an industrial scale. A sensitivity analysis varying H_2_SO_4_ and EtOH recovery rates further demonstrated the critical influence of reagent recovery efficiency on production feasibility, with costs ranging from 9.43–13.96 US$ kg^−1^ at 0% recovery to 1.53–1.80 US$ kg^−1^ at 95% recovery. Nevertheless, these results revealed the potential for using the coarse and fine fractions of BOF sludge as raw material to produce alternative coagulants for water treatment.

## Supplementary Information

Below is the link to the electronic supplementary material.

ESM1 (DOCX 56.6 KB)

## Data Availability

Data will be made available on request.
