# Supplementary material for: Ferric coagulants produced from basic oxygen furnace sludge—Part II: optimization of ferrous sulfate production and application of ferric coagulants in water treatment
Source: Environ Sci Pollut Res Int. 2026 Jul 3;33(21):10607–25. doi: 10.1007/s11356-026-37973-9 (PMC13369228; doi:10.1007/s11356-026-37973-9)
Supplement: Supplementary file 1 — (DOCX 56.6 KB) [file 11356_2026_37973_MOESM1_ESM.docx]

**Ferric coagulants produced from basic oxygen furnace sludge − Part II: Optimization of ferrous sulfate production and application of ferric coagulants in water treatment**

Luisa Cardoso Maia^1,*^, Grazielle Rocha dos Santos^1^, Andressa Rezende Pereira^1^, Yasmim Arantes da Fonseca^1^, Leandro Vinícius Alves Gurgel^2^, Cornélio de Freitas Carvalho^1^

^1^ Environmental Engineering Graduate Program, School of Mines, Federal University of Ouro Preto, Campus Universitário Morro do Cruzeiro, Rua Nove, s/n, Bauxita, 35402-163, Ouro Preto, Minas Gerais, Brazil.

^2^ Department of Chemistry, Institute of Exact and Biological Sciences, Federal University of Ouro Preto, Campus Universitário Morro do Cruzeiro, Rua Quatro, 786, Bauxita, 35402-136, Ouro Preto, Minas Gerais, Brazil.

*Corresponding author. Tel.: +55 31 3559-1725; E-mail address: [luisa.maia@ufop.edu.br](mailto:luisa.maia@ufop.edu.br); [luisacardosomaia@gmail.com](mailto:luisacardosomaia@gmail.com) (L.C. Maia).

**Supplementary Table 1.** 2^4^ experimental design used for ferrous sulfate production from the fine fraction of BOF sludge.

| **Experiment** | ***c*** ^(a)^ **(%)** | ***t*** ^(b)^ **(min)** | ***w*** ^(c)^ **(g)** | ***e*** ^(d)^ **(mL)** | **FeSO_4_·7H_2_O (g)** | **Yield (%)** |
| --- | --- | --- | --- | --- | --- | --- |
| 01 | 10 (−1) | 100 (−1) | 4.01 (−1) | 60 (−1) | 0.58 | 7.02 |
| 02 | 30 (+1) | 100 (−1) | 4.00 (−1) | 60 (−1) | 0.13 | 1.59 |
| 03 | 10 (−1) | 200 (+1) | 4.00 (−1) | 60 (−1) | 2.22 | 26.87 |
| 04 | 30 (+1) | 200 (+1) | 4.00 (−1) | 60 (−1) | 0.06 | 0.74 |
| 05 | 10 (−1) | 100 (−1) | 6.01 (+1) | 60 (−1) | 8.55 | 69.05 |
| 06 | 30 (+1) | 100 (−1) | 6.01 (+1) | 60 (−1) | 4.25 | 34.33 |
| 07 | 10 (−1) | 200 (+1) | 6.00 (+1) | 60 (−1) | 9.78 | 79.07 |
| 08 | 30 (+1) | 200 (+1) | 6.01 (+1) | 60 (−1) | 6.19 | 50.02 |
| 09 | 10 (−1) | 100 (−1) | 4.00 (−1) | 90 (+1) | 6.29 | 76.18 |
| 10 | 30 (+1) | 100 (−1) | 4.01 (−1) | 90 (+1) | 3.17 | 38.45 |
| 11 | 10 (−1) | 200 (+1) | 4.01 (−1) | 90 (+1) | 3.68 | 44.59 |
| 12 | 30 (+1) | 200 (+1) | 4.00 (−1) | 90 (+1) | 0.09 | 1.13 |
| 13 | 10 (−1) | 100 (−1) | 6.01 (+1) | 90 (+1) | 10.03 | 81.02 |
| 14 | 30 (+1) | 100 (−1) | 6.00 (+1) | 90 (+1) | 7.19 | 58.10 |
| 15 | 10 (−1) | 200 (+1) | 6.00 (+1) | 90 (+1) | 10.26 | 82.94 |
| 16 | 30 (+1) | 200 (+1) | 6.00 (+1) | 90 (+1) | 8.55 | 69.12 |
| 17 | 20 (0) | 150 (0) | 5.00 (0) | 75 (0) | 6.37 | 61.80 |
| 18 | 20 (0) | 150 (0) | 5.01 (0) | 75 (0) | 6.09 | 59.06 |
| 19 | 20 (0) | 150 (0) | 5.00 (0) | 75 (0) | 6.06 | 58.78 |

^(a)^ H_2_SO_4_ solution concentration (v/v); ^(b)^ Leaching time; ^(c)^ Waste amount; ^(d)^ Ethanol volume.

**Supplementary Table 2.** Analysis of variance of the linear model of ferrous sulfate process from the fine fraction of BOF sludge (bold value is significant, i.e., *p* < 0,05).

| **Independent variable**: yield (%) | | | | | |
| --- | --- | --- | --- | --- | --- |
| **Source of variation** | **Quadratic Sum** | **Degrees of freedom** | **Mean square** | ***F* value** | ***p-*value** |
| Regression | 14,471.9 | 15 | 964.8 | 208.1 | **0.0005** |
| Residues | 13.9 | 3 | 4.6 |  |  |
| Lack of fit | 8.3 | 1 | 8.3 | 3.0 | 0.2 |
| Pure error | 5.6 | 2 | 2.8 |  |  |
| **Total** | 14,485.9 | 18 |  |  |  |
| **Variation explained** (*R*^2^) = 99.90% / Adjusted variation explained (*R*^2^_adj_) = 99.42% | | | | | |

**Supplementary Table 3.** Analysis of variance of the quadratic model produced by DED for the optimization of ferrous sulfate production (bold value is significant, i.e., *p* < 0,05).

| **Independent variable**: yield (%) | | | | | |
| --- | --- | --- | --- | --- | --- |
| **Source of variation** | **Quadratic Sum** | **Degrees of freedom** | **Mean square** | ***F* value** | ***p-*value** |
| Regression | 10,000.0 | 14 | 714.3 | 9.9 | **0.001** |
| Residues | 575.3 | 8 | 71.9 |  |  |
| Lack of fit | 562.8 | 6 | 93.8 | 15.0 | 0.06 |
| Pure error | 12.5 | 2 | 6.3 |  |  |
| **Total** | 10,575.3 | 22 |  |  |  |
| Variation explained (*R*^2^) = 94.56% / Adjusted variation explained (*R*^2^_adj_) = 85.04% | | | | | |

**Supplementary Table 4.** Thermal decomposition of ferrous sulfate produced from BOF sludge (fine fraction).

| **TG changes** | ***T*_onset_ (ºC)** | ***T*_endset_ (ºC)** | **Mass changes (%)** |
| --- | --- | --- | --- |
| 1st step | 27.4 | 75.1 | 17.1 |
| 2nd step | 75.1 | 151.6 | 19.0 |
| 3rd step | 151.6 | 288.3 | 5.3 |
| 4th step | 288.3 | 641.4 | 30.2 |
| 5th step | 641.4 | 798.4 | 6.1 |
| **Loss on ignition** | | | 77.6 |

**Supplementary Table 5.** Optimization tests for water clarification in high-turbidity samples using FC-I.

| **Jars** | **Initial turbidity (NTU)** | **pH** | **Coagulant dose (mg L^−1^)** | **Final turbidity (NTU)** | **Efficiency (%)** |
| --- | --- | --- | --- | --- | --- |
| 1 | 263 ± 1 | 6.85 ± 0.01 | 5 | 19.7 ± 0.2 | 92.5 ± 0.1 |
| 2 | 230 ± 1 | 6.95 ± 0.01 | 10 | 10.8 ± 0.2 | 95.3 ± 0.1 |
| 3 | 238 ± 7 | 6.55 ± 0.03 | 15 | 5.9 ± 0.5 | 97.5 ± 0.2 |
| 4 | 242 ± 2 | 6.49 ± 0.07 | 20 | 10.9 ± 0.8 | 95.5 ± 0.3 |
| 5 | 233 ± 4 | 6.2 ± 0.1 | 25 | 27 ± 1 | 88.4 ± 0.6 |
| 6 | 223± 1 | 6.37 ± 0.02 | 30 | 20.1 ± 0.6 | 91.0 ± 0.3 |

**Supplementary Table 6.** Optimization tests for water clarification in high-turbidity samples using FC-II.

| **Jars** | **Initial turbidity (NTU)** | **pH** | **Coagulant dose (mg L^−1^)** | **Final turbidity (NTU)** | **Efficiency (%)** |
| --- | --- | --- | --- | --- | --- |
| 1 | 192 ± 1 | 7.07 ± 0.01 | 5 | 21.6 ± 0.8 | 88.8 ± 0.4 |
| 2 | 197 ± 4 | 6.89 ± 0.06 | 10 | 8.9 ± 0.5 | 95.5 ± 0.2 |
| 3 | 194 ± 2 | 6.44 ± 0.03 | 15 | 12.5 ± 0.2 | 93.6 ± 0.1 |
| 4 | 199 ± 1 | 6.7 ± 0.1 | 20 | 9.6 ± 0.5 | 95.2 ± 0.2 |
| 5 | 192 ± 6 | 6.83 ± 0.02 | 25 | 17.8 ± 0.6 | 90.7 ± 0.4 |
| 6 | 193 ± 5 | 6.32 ± 0.05 | 30 | 20.3 ± 0.6 | 89.5 ± 0.4 |

**Supplementary Table 7.** Optimization tests for water clarification in high-turbidity samples using PAC.

| **Jars** | **Initial turbidity (NTU)** | **pH** | **Coagulant dose (mg L^−1^)** | **Final turbidity (NTU)** | **Efficiency (%)** |
| --- | --- | --- | --- | --- | --- |
| 1 | 192 ± 6 | 7.55 ± 0.02 | 5 | 4.9 ± 0.4 | 97.4 ± 0.2 |
| 2 | 172 ± 1 | 7.90 ± 0.01 | 10 | 5.5 ± 0.3 | 96.8 ± 0.2 |
| 3 | 164 ± 4 | 7.85 ± 0.07 | 15 | 9.0 ± 0.1 | 94.5 ± 0.2 |
| 4 | 187 ± 3 | 7.90 ± 0.00 | 20 | 11 ± 2 | 94.1 ± 0.9 |
| 5 | 172 ±1 | 7.64 ± 0.02 | 25 | 9.1 ± 0.7 | 94.7 ± 0.4 |
| 6 | 155 ± 2 | 7.53 ± 0.05 | 30 | 8.4 ± 0.0 | 94.6 ± 0.1 |

**Supplementary Table 8.** Input values considered for economic analysis.

| **Inputs** | | **Units** | **Values** |
| --- | --- | --- | --- |
| H_2_SO_4_ 95-98% P.A. ACS | | US$ L^−1^ | 7.37 ^(a)^ |
| Anhydrous ethanol 99% P.A. ACS | | US$ L^−1^ | 3.83 ^(a)^ |
| H_2_O_2_ 35% P.A. ACS | | US$ L^−1^ | 1.83 ^(a)^ |
| BOFs sludge moisture content | | % | 35% ^(b)^ |
| Water (industrial category > 200 m³) | | US$ L^−1^ | 0.002 ^(c)^ |
| Electricity | | US$ kWh^−1^ | 0.11 ^(d)^ |
| Motor rating input – orbital shaker | | W | 120 |
| Motor rating input – vacuum pump | | W | 85 |
| H_2_SO_4_ recycling | | % | 80 ^(e)^ |
| Ethanol recycling | | % | 80 ^(f)^ |
| ^(a)^ Neon (2021); ^(b)^ Das et al. (2007); ^(c)^ Copasa (2020); ^(d)^ Cemig (2021); ^(e)^ Amaral et al. (2018); ^(f)^ Vigânico (2014). | | |  |

**Supplementary Table 9.** Estimated costs of the acid leaching process of the coarse and fine fractions of BOF sludge.

| **Estimated cost for the first step** – acid leaching | | | | |
| --- | --- | --- | --- | --- |
| **BOF sludge**  **Inputs** | **Coarse fraction** | | **Fine fraction** | |
|  | **Quantity** | **Price (US$)** | **Quantity** | **Price (US$)** |
| Amount of steel waste (g) | 7.00 | 0.00 | 7.00 | 0.00 |
| Water from sludge moisture content (mL kg^-1^) | 2.5 | 0.00 | 2.5 | 0.00 |
| Volume of H_2_SO_4_ 95-97% P.A. ACS (mL) | 2.0 ^(a)^ | 0.02 | 1.3 ^(a)^ | 0.01 |
| Volume of process water (mL) | 45.6 | 0.0001 | 46.3 | 0.0001 |
| Electricity (kWh) | 0.40 | 0.05 | 0.28 | 0.03 |
| **Total cost (US$)** | 0.06 | | 0.04 | |

^(a)^ Considering an 80% acid recovery percentage, as reported by Amaral et al (2018).

**Supplementary Table 10.** Estimated costs of the filtration stage after leaching of the coarse and fine fractions of BOF sludge.

| **Estimated cost for the second step** – filtration | | | | |
| --- | --- | --- | --- | --- |
| **BOF sludge**  **Inputs** | **Coarse fraction** | | **Fine fraction** | |
|  | **Quantity** | **Price (US$)** | **Quantity** | **Price (US$)** |
| Electricity ^(a)^ (kWh) | 0.001 | 0.0002 | 0.001 | 0.0002 |
| **Total cost (US$)** | 0.0002 | | 0.0002 | |

^(a)^ Considering a quantity of steel waste of 7.00 g.

**Supplementary Table 11.** Estimated costs of the crystallization of ferrous sulfate heptahydrate produced from the coarse and fine fractions of BOF sludge.

| **Estimated cost for the third step** – crystallization | | | | |
| --- | --- | --- | --- | --- |
| **BOF sludge**  **Inputs** | **Coarse fraction** | | **Fine fraction** | |
|  | **Quantity** | **Price (US$)** | **Quantity** | **Price (US$)** |
| BOF sludge mass ^(b)^ (g) | 7.00 | 0.00 | 7.00 | 0.00 |
| Volume of anhydrous ethanol ^(b)^ (mL) | 22 ^(a)^ | 0.08 | 24 ^(a)^ | 0.09 |
| **Total cost (US$)** | 0.08 | | 0.09 | |

^(a)^ Considering an 80% ethanol recycling percentage, as reported by Vigânico (2014).

^(b)^ Considering a quantity of steel waste of 7.00 g.

**Supplementary Table 12.** Estimated costs of the filtration of ferrous sulfate crystals produced from the coarse and fine fractions of BOF sludge.

| **Estimated cost for the fourth step** – filtration of ferrous sulfate crystals | | | | |
| --- | --- | --- | --- | --- |
| **BOF sludge**  **Inputs** | **Coarse fraction** | | **Fine fraction** | |
|  | **Quantity** | **Price (US$)** | **Quantity** | **Price (US$)** |
| Electricity ^(a)^ (kWh) | 0.001 | 0.0002 | 0.001 | 0.0002 |
| **Total cost (US$)** | 0.0002 | | 0.0002 | |

^(a)^ Considering a quantity of steel waste of 7.00 g.

**Supplementary Table 13.** Estimated costs of the ferric coagulants produced from BOFs sludge.

| **Estimated cost for the fifth step** – ferrous sulfate oxidation | | | | |
| --- | --- | --- | --- | --- |
| **BOF sludge**  **Inputs** | **Coarse fraction** | | **Fine fraction** | |
|  | **Quantity** | **Price (US$)** | **Quantity** | **Price (US$)** |
| Amount of steel waste (g) | 7.00 | 0.00 | 7.00 | 0.00 |
| FeSO_4_·7H_2_O (g) | 19.6 | 0.144 | 13.1 | 0.133 |
| FeSO_4_·7H_2_O (**US$ kg^−1^**) | 1 kg FS-I | 7.4 **US$ kg^−1^** | 1 kg FS-II | 10.2 **US$ kg^−1^** |
| H_2_O_2_:FeSO_4_·7H_2_O ratio (mL g^−1^) | 0.4 | - | 0.4 | - |
| H_2_O:FeSO_4_·7H_2_O ratio (mL g^−1^) | 1.5 | - | 1.5 | - |
| H_2_SO_4_:FeSO_4_·7H_2_O ratio (mL g^−1^) | 0.05 | - | 0.05 | - |
| Volume of H_2_O_2_ (mL) | 7.84 | 0.014 | 5.22 | 0.010 |
| Volume of H_2_O (mL) | 29.4 | 0.00006 | 19.59 | 0.00004 |
| Volume of H_2_SO_4_ (mL) | 0.98 | 0.007 | 0.65 | 0.005 |
| Total cost (US$) | 0.166 | | 0.148 | |
| Volume of Fe_2_(SO_4_)_3_ produced (mL) | 48.5 | | 32.3 | |
| Mass of Fe_2_(SO_4_)_3_ produced (g) | 59.6 | | 39.7 | |
| **Ferric coagulant FC-I (US$ L^−1^)** | 3.4 | | 4.6 | |
| **Ferric coagulant FC-II (US$ kg^−1^)** | 2.8 | | 3.7 | |

**References**

Amaral MCS, Grossi LB, Ramos RL, Ricci BC, Andrade LH (2018) Integrated UF–NF–RO route for gold mining effluent treatment: From bench-scale to pilot-scale. Desalination 440:111-121. <https://doi.org/10.1016/j.desal.2018.02.030>

Cemig (2021) Tariffs and services values. Energy Company of Minas Gerais (Cemig), Brazil. https://www.cemig.com.br/valores-e-tarifas/tarifas-vigentes/. Accessed 15 December 2021

Companhia de Saneamento de Minas Gerais (Copasa) (2020) ARSAE-MG Resolution Nº 141, June 22, 2020. Belo Horizonte, Brazil: Copasa. https://www.arsae.mg.gov.br/images/documentos/legislacao/2020/Resolucao_141_ReajusteCopasa_2020.pdf. Accessed 12 December 2021

Das B, Prakash S, Reddy PSR, Misra VN (2007) An overview of utilization of slag and sludge from steel industries. Resour Conserv Recy 50:40-57. <https://doi.org/10.1016/j.resconrec.2006.05.008>

Neon Comercial (2021) Products. São Paulo: Neon Comercial. In: https://www.neoncomercial.com.br/produtos/. Accessed 26 June 2021

Vigânico EM (2014): Pilot-scale prototype for the production of ferrous sulfate from pyrite concentrate from a coal mining (in Portuguese), Federal University of Rio Grande do Sul, Porto Alegre, Brazil.
